# Mechanical Regulation Underlies Effects of Exercise on Serotonin-Induced Signaling in the Prefrontal Cortex Neurons

**DOI:** 10.1016/j.isci.2020.100874

**Published:** 2020-01-31

**Authors:** Youngjae Ryu, Takahiro Maekawa, Daisuke Yoshino, Naoyoshi Sakitani, Atsushi Takashima, Takenobu Inoue, Jun Suzurikawa, Jun Toyohara, Tetsuro Tago, Michiru Makuuchi, Naoki Fujita, Keisuke Sawada, Shuhei Murase, Masashi Watanave, Hirokazu Hirai, Takamasa Sakai, Yuki Yoshikawa, Toru Ogata, Masahiro Shinohara, Motoshi Nagao, Yasuhiro Sawada

**Affiliations:** 1 Department of Rehabilitation for Motor Functions, National Rehabilitation Center for Persons with Disabilities, Tokorozawa, Saitama 359-8555, Japan; 2 Department of Veterinary Surgery, Graduate School of Agricultural and Life Sciences, The University of Tokyo, Bunkyo, Tokyo 113-8657, Japan; 3 Division of Advanced Applied Physics, Institute of Engineering, Tokyo University of Agriculture and Technology, Koganei, Tokyo 184-8588, Japan; 4 Department of Assistive Technology, National Rehabilitation Center for Persons with Disabilities, Tokorozawa, Saitama 359-8555, Japan; 5 Research Team for Neuroimaging, Tokyo Metropolitan Institute of Gerontology, Itabashi, Tokyo 173-0015, Japan; 6 Section of Neuropsychology**,** National Rehabilitation Center for Persons with Disabilities, Tokorozawa, Saitama 359-8555, Japan; 7 University of Cincinnati College of Medicine, Cincinnati, OH 45267, USA; 8 Department of Neurophysiology & Neural Repair, Gunma University Graduate School of Medicine, Maebashi, Gunma 371-8511, Japan; 9 Department of Bioengineering, Graduate School of Engineering, The University of Tokyo, Bunkyo, Tokyo 113-8656, Japan; 10 Department of Clinical Research, National Rehabilitation Center for Persons with Disabilities, Tokorozawa, Saitama 359-8555, Japan

**Keywords:** Biological Sciences, Neuroscience, Molecular Neuroscience, Cellular Neuroscience

## Abstract

Mechanical forces are known to be involved in various biological processes. However, it remains unclear whether brain functions are mechanically regulated under physiological conditions. Here, we demonstrate that treadmill running and passive head motion (PHM), both of which produce mechanical impact on the head, have similar effects on the hallucinogenic 5-hydroxytryptamine (5-HT) receptor subtype 2A (5-HT_2A_) signaling in the prefrontal cortex (PFC) of rodents. PHM generates interstitial fluid movement that is estimated to exert shear stress of a few pascals on cells in the PFC. Fluid shear stress of a relevant magnitude on cultured neuronal cells induces ligand-independent internalization of 5-HT_2A_ receptor, which is observed in mouse PFC neurons after treadmill running or PHM. Furthermore, inhibition of interstitial fluid movement by introducing polyethylene glycol hydrogel eliminates the effect of PHM on 5-HT_2A_ receptor signaling in the PFC. Our findings indicate that neuronal cell function can be physiologically regulated by mechanical forces in the brain.

## Highlights

• Mechanical forces regulate brain functions under physiological conditions
• Intracerebral interstitial fluid has mechanical roles in regulating brain functions
• Mechanical impact on the head mediates effects of exercise on the brain
• Fluid shear stress physiologically modulates signaling in nervous cells

## Graphical Abstract

**Figure:**
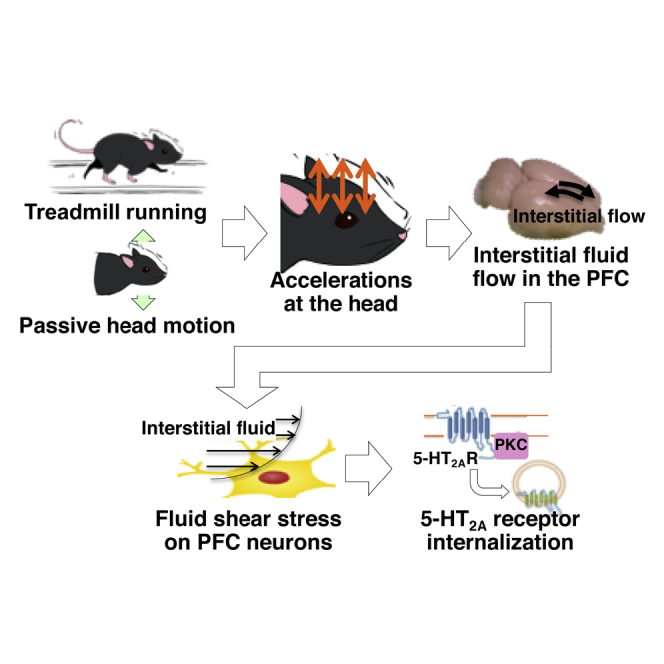

## Introduction

As the phrase “Exercise is Medicine” indicates physical exercise has been widely recognized to be effective in maintaining homeostasis of various tissues and organs. For example, aerobic exercise has been reported to be beneficial as a therapeutic intervention for cardiovascular, metabolic, and musculoskeletal disorders (Haskell et al., 2007). Exercise is also advantageous for functions of the nervous system. Therapeutic effects of exercise on numerous brain-related health problems such as dementia, schizophrenia, depression, and essential hypertension have been demonstrated (Chaar et al., 2015, Kirk-Sanchez and McGough, 2014, van Praag et al., 2014, Wang et al., 2018). However, molecular mechanisms underlying these positive effects of physical exercise on brain functions are poorly understood. Although several factors released from musculoskeletal organs, which include irisin, brain-derived neurotropic factor, and osteocalcin, are associated with the nervous system (Oury et al., 2013, Wrann, 2015), it is enigmatic whether these factors underlie the benefit of exercise with regard to brain functions. Because of this lack of adequate understanding, it is difficult to develop a scientific evidence-based guideline for exercise as a therapeutic/preventative intervention for brain-related disorders.

Among numerous biochemical signals that function in the nervous system, those related to serotonin (5-hydroxytryptamine, herein referred to as 5-HT) play essential roles in regulating emotions and behaviors and are implicated in the aforementioned psychiatric diseases (Berger et al., 2009, Canli and Lesch, 2007, Roth et al., 2004), on which exercise has been proved to have therapeutic effects. Receptors for 5-HT are expressed in various regions of the brain such as the amygdala, hippocampus, and cerebral cortex (Guiard and Di Giovanni, 2015). 5-HT receptor signaling in the prefrontal cortex (PFC), which modulates cortical neuronal activity and oscillation related to emotion and cognition (Puig and Gulledge, 2011), is implicated in psychiatric disorders (Siddiqui et al., 2008). 5-HT receptor subtypes 1A and 2A (5-HT_1A_ and 5-HT_2A_ receptors, respectively) are the two major 5-HT receptors expressed in the PFC (Puig and Gulledge, 2011). 5-HT_1A_ receptor signaling activation leads to the suppression of neuronal activity, whereas 5-HT_2A_ receptor activation provokes neuronal circuit excitability (Puig and Gulledge, 2011). Alteration in 5-HT_2A_ receptor signaling, the absence of which reduces anxiety-like behavior in mice (Weisstaub et al., 2006), is associated with many psychiatric disorders such as depression, schizophrenia, and hallucinogenic phenotypes in humans (Aghajanian and Marek, 1999, Nichols, 2004, Roth et al., 2004). Systemic administration of 2,5-dimethoxy-4-iodoamphetamine, a 5-HT_2A_ receptor agonist, enhances a depressive character, whereas pre-treatment with MDL100907, a 5-HT_2A_ receptor antagonist, eliminates this effect (Diaz and Maroteaux, 2011). Treatment with D-lysergic acid diethylamide, which possesses hallucinogenic potential through massive activation of 5-HT_2A_ receptor signaling in the PFC (Egan et al., 1998), is used as a pharmacological model of psychosis in both human and animal studies (Marek and Aghajanian, 1996, Preller et al., 2018). In contrast to these negative aspects, pharmacological activation of 5-HT_2A_ receptor signaling also positively regulates cognitive functions such as learning and memory. In mice, (4-bromo-3,6-dimethoxybenzocyclobuten-1-yl)methylamine hydrobromide (TCB-2), a selective 5-HT_2A_ receptor agonist, enhances consolidation of object memory when it is administered systemically during a behavioral test for working memory (Zhang et al., 2013). Collectively, 5-HT_2A_ receptor signaling in the PFC supports normal cognitive functions; however, it can mediate emotional or psychotic disorders when it is excessively or aberrantly activated.

Many of physical exercise procedures, particularly aerobic exercises, generate mechanical loads on the head. Signaling of angiotensin II type I receptor, a member of the G-protein-coupled receptor (GPCR) family, is modified ligand independently by mechanical stress (Zou et al., 2004). Although we previously reported that treadmill running caused changes in the distribution and activity of 5-HT_2A_ receptor, another GPCR family member, in spinal cord neurons of rats (Ryu et al., 2018), it remains elusive how exercise modulates 5-HT_2A_ receptor signaling in the nervous system. Because the activity of 5-HT_2A_ receptor is modulated by its internalization, either ligand dependently or ligand independently (Bhattacharyya et al., 2002), we hypothesized that 5-HT_2A_ receptor signaling in the brain might be mechanically regulated by the receptor internalization.

## Results

### Both Treadmill Running and Passive Head Motion Alleviate 5-HT Receptor Signaling in the Brain

**Figure 1:**
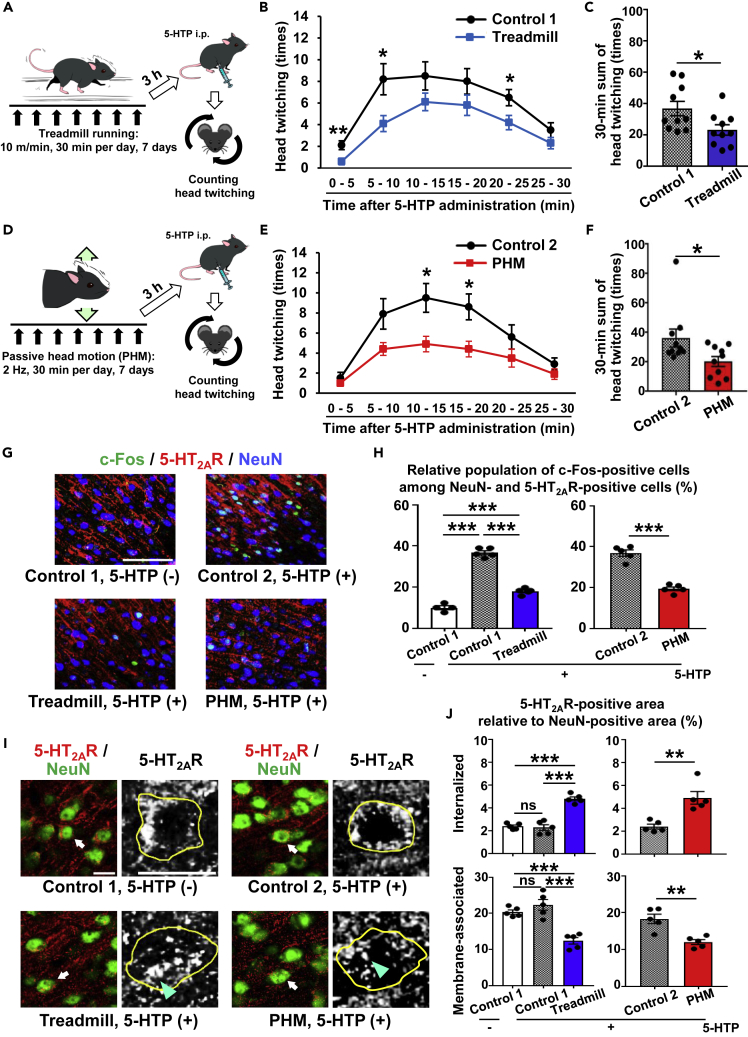
Treadmill Running and Passive Head Motion (PHM) Similarly Modulate Behavioral and Neuronal Response to 5-HT (A) Schematic representation of experimental protocol for analysis of the effects of treadmill running on head-twitch response (HTR). (B and C) Treadmill running alleviated 5-HTP-induced HTR. Count of head twitching in 5-min blocks (B) and for 30 min (C) post-5-HTP administration (p = 0.027, unpaired t test; n = 10 mice for each group). Control 1 in (B, C, G, H, I, and J) represents mice that were placed in the treadmill machine without turning it on (30 min per day, 7 days). (D) Schematic representation of experimental protocol for analysis of the effects of PHM on HTR. PHM (cyclical 5-mm head drop) was applied to generate vertical accelerations of 1.0 × *g* peaks at the heads of mice (2 Hz, 30 min per day, 7 days). (E and F) PHM alleviated 5-HTP-induced HTR. Head twitching was counted as in (B) and (C), respectively (p = 0.035, unpaired t test; n = 10 mice for each group). Control 2 in (E–J) represents mice that were anesthetized and placed in a prone position with their heads on the platform that was left unoscillated (30 min/day, 7 days). (G) Micrographic images of anti-c-Fos (green), anti-5-HT_2A_ receptor (red), and anti-NeuN (blue) immunostaining of the PFC of mice administered 5-HTP (or vehicle) after a week of daily treadmill running or PHM. Scale bar, 100 μm. Images are representative of four to five mice. (H) Both treadmill running and PHM decreased c-Fos expression in 5-HT_2A_ receptor-positive neurons of mouse PFC. Relative population (%) of c-Fos-positive cells of 300 NeuN- and 5-HT_2A_ receptor-positive cells is shown (left chart: p < 0.001, one-way ANOVA with post hoc Bonferroni test; right chart: p < 0.001, unpaired t test; n = 4 mice for columns 1, n = 5 mice for columns 2 to 5). (I) Micrographic images of anti-5-HT_2A_ receptor (5-HT_2A_R; red) and anti-NeuN (green) immunostaining of the PFC of mice injected with 5-HTP (or vehicle) after a week of daily treadmill running or PHM. Higher-magnification images of anti-5-HT_2A_ receptor immunostaining of arrow-pointed cells are presented with a gray scale. Yellow lines indicate the margins of somas outlined by NeuN-positive signals, and cyan arrowheads point to internalized anti-5-HT_2A_ receptor immunosignals. Scale bars, 20 μm. Images are representative of five mice. (J) Quantification of internalized and membrane-associated 5-HT_2A_ receptor-positive area relative to NeuN-positive area in mouse PFC. Thirty-five to forty NeuN-positive neuronal somas were analyzed for each mouse (Internalized: left chart, p < 0.001, one-way ANOVA with post hoc Bonferroni test; right chart, p = 0.0027, unpaired t test; Membrane-associated: left chart, p < 0.001, one-way ANOVA with post hoc Bonferroni test; right chart, p = 0.0025, unpaired t test; n = 5 mice for each group). Data for 5-HTP(+) samples in (G–J) were obtained from mice infused with 4% paraformaldehyde/PBS immediately after HTR test shown in (B, C, E, and F). Data are represented as means ± SEM. *p < 0.05, **p < 0.01, ***p < 0.001; ns, not significant. See also Figures S1, S2, S3, and S4.

We first examined whether treadmill running at a modest velocity (∼10 m/min for mice and ∼20 m/min for rats), a typical experimental intervention to test effects of physical exercise in rodents (Kim et al., 2010, Li et al., 2013), modulated 5-HT_2A_ receptor signaling in the brain. To this end, we quantitatively analyzed the head-twitch response (HTR), a hallucinogenic behavioral reaction that represents 5-HT_2A_ receptor activation in the PFC neurons of rodents (Canal and Morgan, 2012, Halberstadt and Geyer, 2013), induced by administration of 5-hydroxytryptophan (5-HTP), the precursor to 5-HT (Figures S1A and S1B). We found that a week of treadmill running of mice (10 m/min, 30 min per day; see Figure 1A) significantly decreased HTR (Figures 1B and 1C), representing a suppressive effect of exercise on 5-HT_2A_ receptor activation in the PFC neurons. Based on our hypothesis that gravity-derived mechanical perturbations on the brain might underpin the effects of physical exercise, we first measured the acceleration generated at the head during treadmill running. Although we examined mouse HTR because of the ease and reliability of quantitative analysis due to frequent and immediate head twitching of mice after 5-HTP administration (Bedard and Pycock, 1977, Goodwin and Green, 1985), we used rats to analyze mechanical elements related to this study. This was because the larger body size of rats enabled us to utilize various experimental tools required for analysis of physical matters and factors. We observed that the peak magnitude of the vertical acceleration generated at the rats' heads (*z* axis in Figure S1C) during treadmill running (20 m/min) was ∼1.0 × *g* (Figures S1D and S1E). We therefore set up our custom-designed “passive head motion” (hereafter referred to as PHM) system to produce 1.0 × *g* of vertical acceleration peaks at the heads of rodents (mice and rats) to be tested (Figures S1D and S1E). Application of PHM to mice under isoflurane anesthesia (2 Hz, 30 min per day, 7 days; see Figure 1D) led to a decrease in HTR, similar to that after treadmill running (compare Figures 1B and 1C with Figures 1E and 1F). Mice that underwent PHM exhibited neither apparent alert problems nor detrimental consequences on behavioral activity. Collectively, we conclude that PHM and treadmill running have a comparable effect on 5-HT_2A_ receptor signaling in the PFC of rodents.

We then examined how treadmill running and PHM modulated 5-HT_2A_ receptor signaling in mouse PFC neurons. To do so, we conducted immunostaining of brain tissue sections. We observed an increase in c-Fos expression, which is downstream of 5-HT_2A_ receptor activation (Gonzalez-Maeso et al., 2007), in PFC neurons of mice after 5-HTP injection when compared with those of vehicle (saline)-injected control mice (compare top two images in Figure 1G and columns 1 and 2 in Figure 1H). Consistent with the suppressive effect of daily treadmill running or PHM (daily 30 min, 7 days) on HTR (Figures 1A–1F), both interventions down-regulated 5-HTP-induced c-Fos expression in the PFC neurons (bottom two images in Figure 1G, and compare columns 2 versus 3 and 4 versus 5 in Figure 1H). Notably, our quantitative analysis of 5-HT_2A_ receptor distribution in neuronal cells (Figures S2A–S2D; see Methods) revealed that both treadmill running and PHM significantly increased 5-HT_2A_ receptor internalization in mouse PFC neurons (Figures 1I and 1J). In contrast, the expression of 5-HT_2A_ receptor (mRNA and protein) in mouse PFC was not significantly changed by treadmill running or PHM (Figures S2E and S2F). Considering that 5-HT_2A_ receptor expression is highly neuron specific in rodent cerebral cortex (Cornea-Hebert et al., 1999), neither treadmill running nor PHM appears to significantly alter 5-HT_2A_ receptor expression in the PFC neurons. These findings suggest that 5-HT_2A_ receptor internalization, rather than decreased expression, is involved in the suppression of 5-HT_2A_ receptor signaling in PFC neurons by treadmill running or PHM.

Both suppression of HTR and 5-HT_2A_ receptor internalization in the PFC neurons were observed even 72 h after the last bout of 1-week daily treadmill running of mice (10 m/min, 30 min per day) (Figures S3A–S3E). In contrast, neither of them was significant 7 days after the last treadmill running (Figures S3F–S3J). Furthermore, the effects of 1-week daily PHM (2 Hz, 30 min per day) on HTR and 5-HT_2A_ receptor internalization in the PFC neurons were observed 72 h (Figures S4A–S4E), but not 7 days (Figures S4F–S4J), after its last bout. These results indicate that the effects of 1-week daily treadmill running or PHM last longer than 72 h, but shorter than 7 days. The apparent consistency between the longer-term effects of treadmill running and PHM (compare Figures S3 and S4) agrees with the mechanistic correlation between HTR suppression and 5-HT_2A_ receptor internalization in the PFC neurons and supports the relevance of PHM to treadmill running.

### PHM Generates Low-Amplitude Intracerebral Pressure Waves and Induces Interstitial Fluid Flow in the PFC

**Figure 2:**
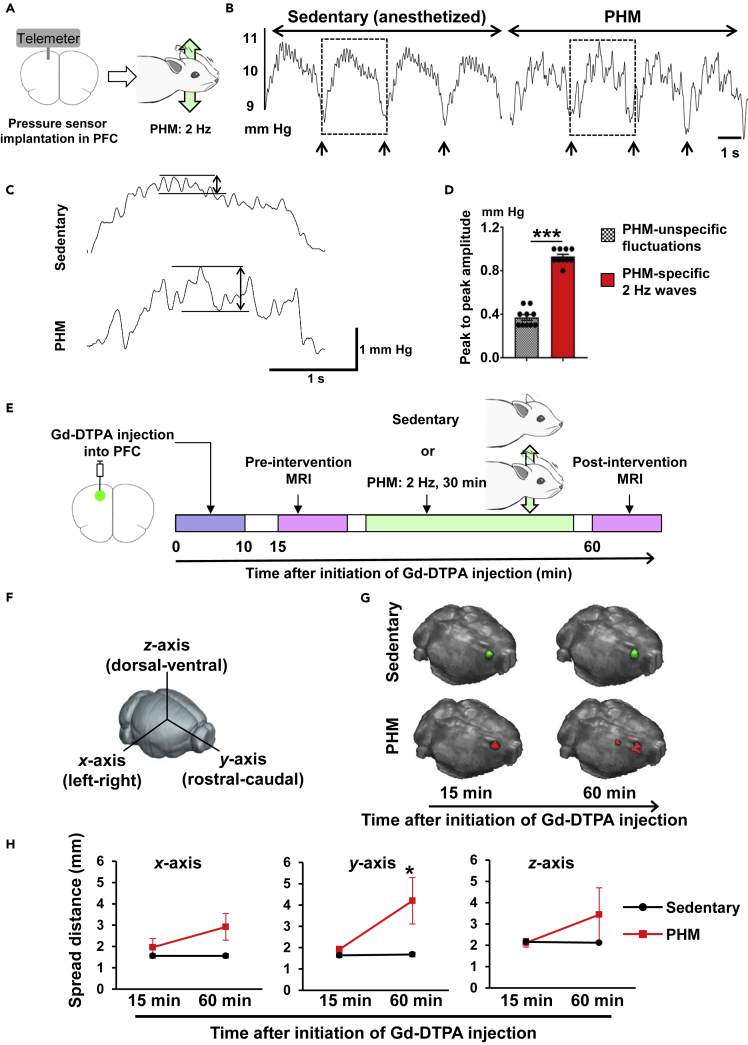
PHM Generates Intracerebral Pressure (ICP) Waves of Low Amplitude, but Facilitates Cerebral Interstitial Fluid Movement (Flow) in the PFC (A–D) PHM generated ∼1 mm Hg ICP changes. (A) Schematic representation of ICP measurement at rats' PFC. (B) Representative ICP waves recorded in rats' PFC during sedentary condition and PHM. Arrows indicate the time points of transition from inhalation to exhalation detected by simultaneous respiration monitoring. Scale bar, 1 s. Images are representative of two independent experiments with similar results. (C) Respiration-unsynchronized ICP changes. Respiration-synchronized ICP waves indicated by rectangles in (B) are presented with higher magnification. Right-angled scale bar, 1 s/1 mm Hg. Note that 2-Hz ICP waves indicated by a two-headed arrow were specifically generated during PHM. (D) Magnitude of PHM-specific and PHM-unspecific ICP changes unsynchronized with respiration. Peak-to-peak magnitudes indicated by two-headed arrows in (C) were quantified. Data are represented as means ± SEM. ***p < 0.001 (unpaired t test, 10 segments analyzed for each). (E–H) PHM facilitates cerebral interstitial fluid movement (flow). (E) Schematic representation of experimental protocol for magnetic resonance imaging analysis of Gd-DTPA injected in rats' PFC. (F) Definition of x (left-right), y (rostral-caudal), and z (dorsal-ventral) axes used in this study. (G) Representative Gd-DTPA spreading presented on a surface-rendered brain. Gd-DTPA clusters are indicated by green (sedentary) and red (PHM). Images are representative of five rats. (H) Quantification of Gd-DTPA spreading along each axis. Red line: PHM (n = 5), black line: sedentary (n = 5). Data are represented as means ± SEM. *p < 0.05 (x axis: p = 0.065, y axis: p = 0.049, z axis: p = 0.33, unpaired t test). See also Figures S5 and S6, and Table S1.

Although vigorous deformations such as stretching of neurons likely have damaging effects (e.g., brain contusion or traumatic brain injury), the brain is not a rigid organ. Therefore, minimal deforming forces or stress distribution changes are possibly produced in the brain under physiological conditions. To analyze the physical effects that PHM produced in the brains of rodents, we measured the local pressure in rats' PFC using a telemetry pressure sensor (Figure 2A). We found that PHM generated pressure waves (changes) with ∼1 mm Hg peak amplitude (Figures 2B–2D). Hydrostatic pressure of this magnitude (∼1.3 cmH_2_O) is unlikely to initiate mechanosensing signaling in cells (Tworkoski et al., 2018). Assuming an analogy with osteocytes embedded in bones, the function of which is known to be modulated by interstitial fluid flow-derived shear stress (Klein-Nulend et al., 2013), we speculated that minimal stress distribution changes might generate interstitial fluid flow in the brain, resulting in shear stress-mediated regulation of neuronal functions.

To measure the PHM-induced interstitial fluid movement in the brain, we injected gadolinium-diethylenetriamine pentaacetic acid (Gd-DTPA), an extracellular fluid contrast agent, into the PFC of anesthetized rats (Figure 2E) and tracked its distribution with magnetic resonance imaging (Figures S5A and S5B). We found that PHM significantly promoted Gd-DTPA spreading in the rostral-caudal (*y* axis, Figure 2F) direction (Figures 2G and 2H). In contrast, PHM did not significantly affect the left-right and dorsal-ventral spreading (x and z axes, Figure 2F) of Gd-DTPA (Figures 2G and 2H), suggesting that PHM may enhance interstitial fluid flow in the PFC in a defined direction, rather than isotropically. Our simulative calculation suggests that PHM subjected PFC neurons to interstitial fluid flow-derived shear stress with an average magnitude of 0.86–3.9 Pa (Table S1). Fluid shear stress (FSS) of this magnitude is known to modify the physiological function of astrocytes (Maneshi et al., 2017), the most abundant type of cells distributed in the brain.

Based on our observation of anisotropic movement of intracerebral interstitial fluid induced by PHM (Figures 2G and 2H), we tested PHM in the directions other than the vertical one. One-week daily PHM (30 min per day) in the left-right direction (Figure S6A, top), which selectively produced 1.0 × *g* of x axis acceleration peaks at rodents' heads (Figures S1C and S6A, bottom), suppressed HTR (Figures S6B and S6C) and induced 5-HT_2A_ receptor internalization in the PFC neurons (Figures S6D and S6E) of mice. In contrast, PHM in the rostral-caudal direction (Figure S6F, top), which produced 1.0 × *g* of y axis acceleration peaks (Figures S1C and S6F, bottom), neither decreased HTR nor induced 5-HT_2A_ receptor internalization in the PFC neurons of mice (Figures S6G-S6J). These results may relate to the directional selectivity (or anisotropy) in intracerebral interstitial fluid movement (Figures 2G and 2H) and agree with the mechanistic correlation between PHM-induced suppression of HTR and 5-HT_2A_ receptor internalization in the PFC neurons of mice.

### FSS on Neuronal Cells Induces 5-HT_2A_ Receptor Internalization

**Figure 3:**
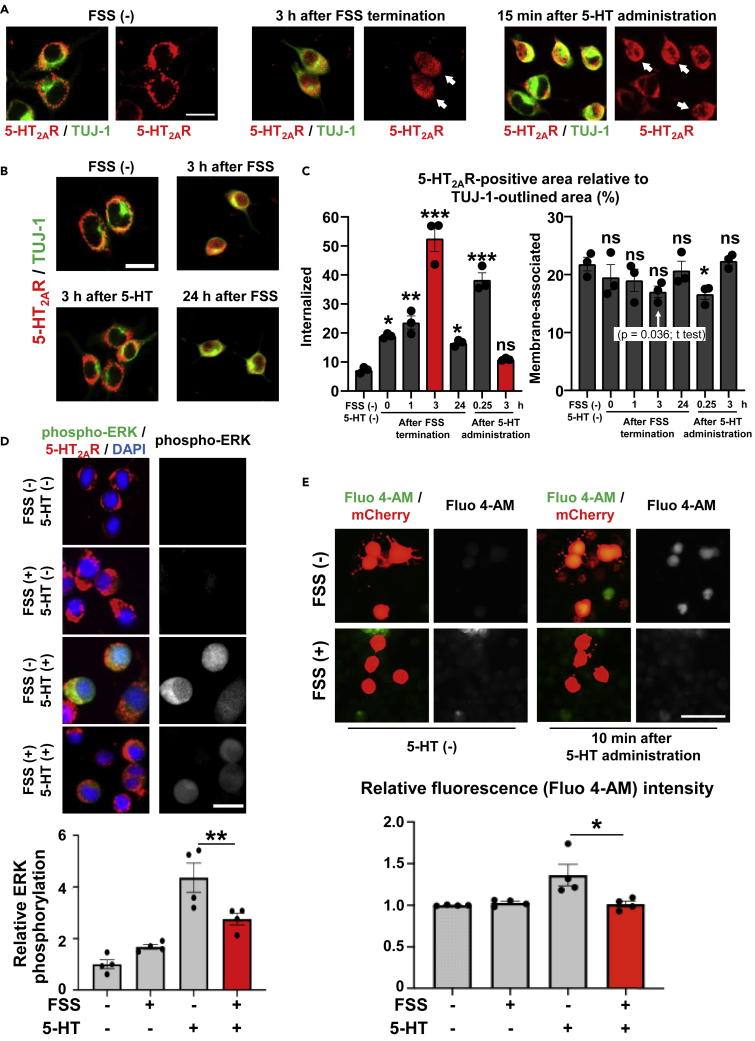
Fluid Shear Stress (FSS) Internalizes 5-HT_2A_ Receptor Expressed in Neuro2A Cells and Modulates Their Responses to 5-HT (A) 5-HT_2A_ receptor was internalized after FSS. Neuro2A cells grown in a poly-D-lysine-coated dish were subjected to pulsatile FSS (average 0.91 Pa, 0.5 Hz, 30 min) or treated with 5-HT (10 μM), fixed, and stained for 5-HT_2A_ receptor (5-HT_2A_R; red). To define their soma outlines, co-immunostaining of TUJ-1 was conducted (green). Left, control; middle, 3 h after the termination of FSS; right, 15 min after 5-HT administration. Arrows point to cells with apparent 5-HT_2A_ receptor internalization. Scale bar, 20 μm. Images are representative of three independent experiments with similar results. (B and C) 5-HT_2A_ receptor internalization lasted longer after FSS, when compared with after 5-HT administration. (B) Micrographic images of Neuro2A cells, with and without FSS application, stained for 5-HT_2A_ receptor (5-HT_2A_R; red) and TUJ-1 (green). Scale bar, 20 μm. Images are representative of three independent experiments with similar results. (C) Quantification of internalized and membrane-associated 5-HT_2A_ receptor-positive area relative to TUJ-1-outlined area in Neuro2A cells. Internalized and membrane-associated 5-HT_2A_ receptor-positive immunosignals were defined as in Figure S2D and quantified as in Figure 1J (left chart: FSS, p < 0.0001 for ANOVA, p = 0.014 for 0 h, p = 0.0016 for 1 h, p < 0.0001 for 3 h, p = 0.049 for 24 h. 5-HT, p < 0.0001 for ANOVA, p < 0.0001 for 0.25 h, p = 0.20 for 3 h. Right chart: FSS, p = 0.36 for ANOVA, p = 0.73 for 0 h, p = 0.58 for 1 h, p = 0.19 for 3 h, p = 0.97 for 24 h. 5-HT, p = 0.010 for ANOVA, p = 0.016 for 0.25 h, p = 0.89 for 3 h, one-way ANOVA with post hoc Dunnett's test; 20 cells analyzed in each sample, n = 3 for each group). (D) FSS alleviated 5-HT-induced ERK phosphorylation in neuronal cells. Neuro2A cells were either left unexposed or exposed to pulsatile FSS (average 0.91 Pa, 0.5 Hz, 30 min). Three hours after FSS termination, cells were treated with 5-HT (10 μM) for 15 min, fixed and stained for 5-HT_2A_ receptor (5-HT_2A_R; red), phospho-ERK (green), and DAPI (blue). Top, micrographic images. Scale bar, 25 μm. Bottom, Quantification of anti-phospho-ERK immuno-intensity: signal intensity of anti-phospho-ERK immunostaining was quantified using “auto-threshold” of ImageJ, and immuno-intensity was calculated by referring the cumulated intensity values to the total positive signal area and scaled with the mean of the control sample (FSS-, 5-HT-) set at 1 (p = 0.0058, one-way ANOVA with post hoc Bonferroni test, 50 cells analyzed in each sample, n = 4 for each group). (E) FSS attenuated 5-HT-induced increase in intracellular Ca^2+^ concentration. Neuro2A cells were either left unexposed or exposed to FSS (average 0.91 Pa, 0.5 Hz, 30 min). Three hours after the termination of FSS, cells were treated with 5-HT (10 μM) for 10 min and subjected to measurement of intracellular Ca^2+^ concentration using Fluo 4-AM as described in Methods. Top, micrographic images. Scale bar, 50 μm. Bottom, intracellular Ca^2+^ concentration represented as relative fluorescence intensity with the mean fluorescence value from cells before 5-HT administration set as 1 (p = 0.0138, one-way ANOVA with post hoc Bonferroni test; 50 cells analyzed in each sample, n = 4 for each group). Data are represented as means ± SEM. *p < 0.05, **p < 0.01, ***p < 0.001; ns, not significant. See also Figure S2.

5-HT_2A_ receptor internalization was commonly observed in PFC neurons of mice after treadmill running and PHM (Figures 1I, 1J, S3D, S3E, S4D, S4E, S6D and S6E). Therefore, we postulated a common regulatory mechanism underlying this internalization and hypothesized that 5-HT_2A_ receptor might be internalized in PFC neurons as a cellular response to mechanical forces generated by the cyclical accelerations with 1.0 × *g* peaks (Figures S1D, S1E and S6A, bottom). To test this hypothesis, we conducted *in vitro* experiments to examine whether 5-HT_2A_ receptor expressed in cultured neuronal cells was internalized in response to FSS. Based on our simulation mentioned above, we applied pulsatile FSS with an average magnitude of 0.91 Pa to Neuro2A cells, which exhibit neuronal phenotypes and morphology (Goshima et al., 1993, Yun et al., 2013), using a system we previously reported (Yoshino et al., 2013). Similar to what was observed after 5-HT administration, FSS application (0.91 Pa, 0.5 Hz, 30 min) caused internalization of 5-HT_2A_ receptors expressed in Neuro2A cells (Figure 3A). However, FSS-induced 5-HT_2A_ receptor internalization appeared different from that after 5-HT administration. As shown in Figures 3B and 3C, 5-HT_2A_ receptor internalization increased incrementally up to 3 h after cessation of FSS (3.5 h after initiation of FSS), whereas it became insignificant 3 h after 5-HT administration. Notably, 5-HT_2A_ receptor internalization remained significantly enhanced even 24 h after FSS application (Figure 3C).

The short duration of ligand-dependent (i.e., 5-HT-induced) 5-HT_2A_ receptor internalization in Neuro2A cells is consistent with the lack of significant changes regarding 5-HT_2A_ receptor internalization in the PFC neurons between mice with and without 5-HTP injection that was carried out >30 min before the transcardial paraformaldehyde infusion (compare columns 1 and 2 in Figure 1J). These findings suggest that the mechanisms of 5-HT_2A_ receptor shuttling/recycling are at least partially distinct between post-FSS application and post-5-HT administration.

Consistent with the 5-HT_2A_ receptor internalization, we observed via immunostaining analysis that FSS alleviated 5-HT-induced phosphorylation of extracellular signal-regulated kinase (ERK) (Figure 3D) and increase in intracellular Ca^2+^ concentration (Figure 3E) in Neuro2A cells. Collectively, these results indicate that FSS desensitizes Neuro2A cells to 5-HT by inducing prolonged 5-HT_2A_ receptor internalization.

### PKCγ Is Responsible for FSS-Induced 5-HT_2A_ Receptor Internalization in Neuro2A Cells

**Figure 4:**
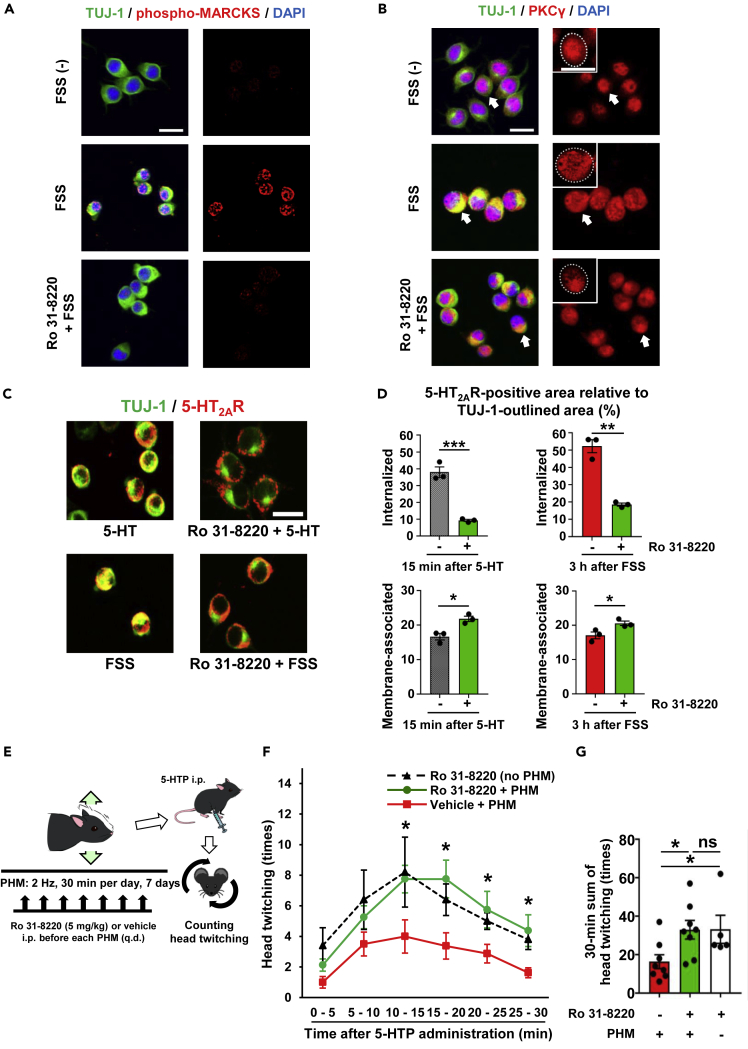
PKC Is Involved in Both FSS-Induced 5-HT_2A_ Receptor Internalization *In Vitro* and PHM-Attenuated HTR *In Vivo* (A and B) MARCKS was phosphorylated in neuronal cells after FSS, depending on PKC activity. Neuro2A cells, either left unexposed or exposed to FSS (average 0.91 Pa, 0.5 Hz, 30 min) with and without PKC inhibitor pretreatment (Ro 31-8220; 4 μM, 1 h), were subjected to anti-TUJ-1 (green) and anti-phospho-MARCKS (red in A) or anti-PKCγ (red in B) immunostaining. Nuclei were stained with DAPI. Higher-magnification images of anti-PKCγ immunostaining of arrow-pointed cells are presented with cell margins (white dashed lines) as insets (B). Scale bars, 20 μm. Images are representative of three independent experiments with similar results. (C) PKC inhibition hampered both 5-HT- and FSS-induced 5-HT_2A_ receptor internalization. Neuro2A cells with combinations of Ro 31-8220 pretreatment and 5-HT administration (10 μM, 15 min) or FSS application (average 0.91 Pa, 0.5 Hz, 30 min) were fixed and stained for 5-HT_2A_ receptor (5-HT_2A_R; red) and TUJ-1 (green). Scale bars, 20 μm. Images are representative of three independent experiments with similar results. (D) Quantification of 5-HT_2A_ receptor internalization (top left chart: p < 0.001, top right chart: p = 0.001, bottom left chart: p = 0.013, bottom right chart: p = 0.041, unpaired t test; 20 cells analyzed in each sample, n = 3 for each group). (E) Schematic representation of the experimental protocol for PHM with PKC inhibition. Ro 31-8220 or vehicle was injected just before each bout of PHM. (F and G) PKC inhibition nullified the effect of PHM on 5-HTP-induced HTR. Head twitching was counted as in Figures 1B and 1C. (F) Count of head twitching in 5-min blocks. Asterisks (*) indicate statistical significance between PHM with (green line) and without (red line) Ro 31-8220 (10–15 min: p = 0.019, 15–20 min: p = 0.012, 20–25 min: p = 0.049, 25–30 min: p = 0.024, unpaired t test; n = 8 mice for each group), whereas there were no significant differences at any time point between Ro 31-8220 with (green line) and without (black line) PHM (p > 0.05, unpaired t test; n = 5 mice for group of Ro 31-8220 without PHM). (G) Total count of head twitching for 30 min after 5-HTP administration (column 1 versus 2: p = 0.016, column 1 versus 3: p = 0.041, column 2 versus 3: p = 0.98, unpaired t test). Data are represented as means ± SEM. *p < 0.05, **p < 0.01, ***p < 0.001; ns, not significant. See also Figures S2 and S7.

We next looked into whether FSS-induced desensitization of Neuro2A cells to 5-HT was relevant to the attenuation of HTR by PHM. It has been reported that HTR is up-regulated in mice that are genetically defective in protein kinase Cγ (PKCγ) (Bowers et al., 2006), the major PKC subtype expressed in neuronal cells (Saito and Shirai, 2002). Furthermore, PKC, which can be activated by FSS (Kroll et al., 1993), is involved in GPCR internalization in various types of cells (Chapell et al., 1998, Liles et al., 1986, Park et al., 2009). We therefore examined whether PKCγ was involved in 5-HT_2A_ receptor internalization after the application of FSS *in vitro*. Our immunostaining analysis of Neuro2A cells revealed that FSS enhanced phosphorylation of myristoylated alanine-rich protein kinase C substrate (MARCKS), a major PKC substrate (Hartwig et al., 1992) (compare top and middle rows in Figure 4A). However, FSS-induced MARCKS phosphorylation was hardly observed in Neuro2A cells pre-treated with a PKC inhibitor, Ro 31-8220 (bottom row in Figure 4A). In addition, FSS application decreased nuclear distribution of PKCγ, which relates to its activation in cultured neuronal cells (Menard et al., 2013) (Figure 4B). Furthermore, PKC inhibition significantly attenuated 5-HT_2A_ receptor internalization in Neuro2A cells after 5-HT administration or FSS application (Figures 4C and 4D). These results suggest that PKC activation is responsible for FSS-induced desensitization of Neuro2A cells to 5-HT.

### Administration of a PKC Inhibitor Eliminates the PHM Effects on the PFC Neurons

We then tested if PKC activation was involved in 5-HT_2A_ receptor internalization *in vivo*, as indicated in the suppressive effect of PHM on HTR. When we injected Ro 31-8220 (5 mg/kg, intraperitoneally) before each bout of daily PHM for a week (Figure 4E), there was an increase in HTR after 5-HTP administration, nullifying the effects of PHM on HTR (Figures 4F and 4G). Histologically, the effects of PHM on c-Fos expression and 5-HT_2A_ receptor internalization in the PFC neurons were significantly reduced by Ro 31-8220 pre-administration (Figure S7). All in all, we conclude that PKCγ activation is at least partly responsible for FSS-induced desensitization of Neuro2A cells to 5-HT *in vitro* as well as the suppressive effect of PHM on HTR after 5-HTP administration *in vivo*. The relatively long duration of FSS-induced 5-HT_2A_ receptor internalization in Neuro2A cells (Figure 3C) poses a possibility of cumulative effects of FSS applied repeatedly. Therefore, our findings support the notion that FSS-induced desensitization of Neuro2A cells *in vitro* is relevant to the suppression of HTR by PHM *in vivo*. Mechanosensitive ion channels that are responsible for FSS-induced increase in intracellular Ca^2+^ (Hyman et al., 2017) may mediate FSS-dependent PKC activation (Berk et al., 1995), but the molecular events upstream of PKC activation in response to FSS remain to be determined.

### PHM Neither Decreases HTR nor Increases 5-HT_2A_ Receptor Internalization when Interstitial Fluid Movement Is Hindered by Hydrogel Introduction in the PFC

**Figure 5:**
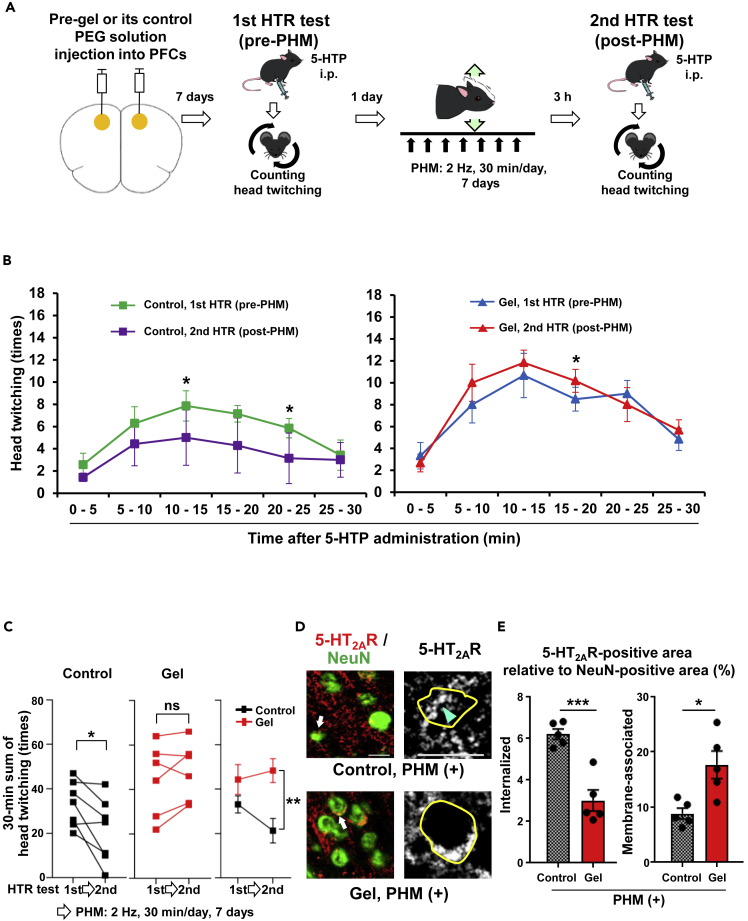
Hydrogel Introduction Modulates Interstitial Fluid Movement and Eliminates Suppressive Effects of PHM on 5-HT_2A_ Receptor Signaling in Mouse PFC Neurons (A) Schematic representation of experimental protocol for analysis of the effects of PHM on HTR with and without PEG hydrogel introduction in mice PFC. PHM was applied daily for 7 days between the first and the second HTR tests. (B and C) Hydrogel introduction in the PFC eliminates the decreasing effects of PHM on HTR. Head twitching was counted as in Figures 1B and 1C (n = 7 mice for control group, n = 6 mice for Gel group). (B) Count of head twitching in 5-min blocks after 5-HTP administration to mice injected with control (left chart, 10–15 min: p = 0.035, 20–25 min: p = 0.042, paired t test) or pre-gel PEG solution (right chart, 15–20 min: p = 0.042, paired t test). (C) Results of the first and second HTR tests are shown by total count of head twitching for 30 min after 5-HTP administration: individual mice (left chart: p = 0.021, center chart: p = 0.22, paired t test) and each group (right chart, first HTR: p = 0.15, second HTR: p = 0.0017, two-way ANOVA with post hoc Bonferroni test). (D) Micrographic images of anti-5-HT_2A_ receptor (5-HT_2A_R; red) and anti-NeuN (green) immunostaining of PFC, either with or without hydrogel introduction, of mice subjected to PHM. Histological samples of the PFC were prepared immediately after the second HTR tests. Higher-magnification images of anti-5-HT_2A_ receptor immunostaining of arrow-pointed cells are presented with a gray scale. Yellow lines indicate the margins of somas outlined by NeuN-positive signals, and the cyan arrowhead points to internalized anti-5-HT_2A_ receptor immunosignals. Scale bars, 20 μm. Images are representative of five mice. (E) Quantification of internalized and membrane-associated 5-HT_2A_ receptor-positive areas relative to NeuN-positive area in mouse PFC. Thirty-five to forty NeuN-positive neuronal somas were analyzed for each sample (left chart: p < 0.001, right chart: p = 0.011, unpaired t test; n = 5 mice for each group). Data are represented as means ± SEM. *p < 0.05, **p < 0.01, ***p < 0.001; ns, not significant. See also Figures S2 and S8.

To examine whether interstitial fluid movement mediated the effects of PHM on the 5-HT_2A_ receptor signaling in PFC neurons, we modulated interstitial fluid dynamics in mouse PFC and conducted PHM experiments. To this end, we gelled the interstitial fluid *in situ* and deprived its fluidity (Figures S8A–S8C) by intracerebrally injecting mutually reactive polyethylene glycol (PEG) gel precursor (pre-gel) solutions (Figure 5A), whose biocompatibility has been confirmed previously (Hayashi et al., 2017). Injection of the pre-gel solutions pre-mixed just before use rendered them spread over mouse PFC and gelled the interstitial fluid *in situ* (Figure S8A). Consistent with our previous observation that gelation only inhibits the fluidity of the fluid but does not restrict the diffusion of small molecules inside the gel (Fujiyabu et al., 2018), hydrogel introduction did not cause apparent delays in HTR after 5-HTP injection (Figure S8D), indicating the rapid access of 5-HT to PFC neurons.

Although 1-week daily PHM decreased HTR in mice injected with the control PEG solution, hydrogel introduction in the PFC eliminated this decreasing effect of PHM (Figures 5B and 5C). Hydrogel introduction alone appeared to slightly increase HTR when compared with the control PEG injection (see first HTR test, Figure 5C, right chart), whereas PHM made the difference significant (see second HTR test, Figure 5C, right chart). This is consistent with the notion that interstitial fluid movement is involved in the suppressive effect of PHM on HTR. Furthermore, hydrogel introduction eliminated the increasing effect of PHM on 5-HT_2A_ receptor internalization in mouse PFC neurons (Figures 5D and 5E). In addition to the aforementioned rapid solute diffusivity through the hydrogels, the expression level of 5-HT_2A_ receptor (Figures S8E and S8F), neuronal survival and apoptosis (Figures S8G and S8H), and overall cell apoptosis (Figure S8I) in mouse PFC were not altered by hydrogel introduction. Therefore it is unlikely that the increasing effects of hydrogel introduction on HTR and membrane association of 5-HT_2A_ receptor resulted from decreased cell viability caused by impaired nutrient supply or removal of metabolic wastes. Collectively, hydrogel introduction in mouse PFC appears to eliminate PHM effects by hindering interstitial fluid movement, indicating the relevance of FSS-induced desensitization of Neuro2A cells to 5-HT *in vitro* to the suppression of HTR by PHM *in vivo*.

## Discussion

Here, we demonstrate that mechanical perturbation that reproduces mechanical impact on the head during walking or light jogging modulates 5-HT_2A_ receptor signaling in the PFC of rodents. Many aerobic exercises, including walking and running, involve impact-generating bodily actions creating sharp accelerations at the head upon foot contacting with the ground. Therefore, their beneficial effects as therapeutic/preventative procedures for a variety of diseases and health disorders may rely at least partly on modest changes in mechanical stress distribution in the brain, which may prompt optimal FSS on cerebral neurons. It is possible that such mechanical impact concomitant with walking/running underlies positive effects of exercise. Our findings suggest that the effects of walking and running on emotional regulation (Edwards et al., 2017) may involve serotonergic modulations in the cerebral cortex induced by mechanical impact on the head.

The amplitude of intracerebral pressure waves generated by PHM (frequency, 2 Hz) reproducing the peak acceleration magnitude at the heads induced by treadmill running was low (∼1 mm Hg) when compared with that produced in synchronization with respiration (amplitude, ∼2 mm Hg; frequency, ∼0.5 Hz) (Figures 2B–2D). Intracerebral pressure waves with lower amplitude (∼0.4 mm Hg) were also observed independent of respiration and PHM (Figures 2B–2D). Judging from the frequency of ∼0.4 mm Hg pressure waves (∼6.6 Hz), they are likely to derive from arterial pulsation or heartbeat. During the intracerebral pressure measurement, rats, either left sedentary or subjected to PHM, kept on breathing with their hearts beating. Therefore, neither ∼2 mm Hg nor ∼0.4 mm Hg intracerebral pressure waves could account for the PHM-specific interstitial fluid movement (Figures 2G, 2H, S5A, and S5B). Given the structural organization of interstitium that has been recently reported in many organs (Benias et al., 2018), the main element behind the physics of PHM-induced intracerebral interstitial fluid flow may not be the magnitude of pressure changes. Instead, other factors such as frequency and direction of accelerations may be critical for interstitial fluid flow generation in the PFC. In line with this notion, PHM effects on HTR and 5-HT_2A_ receptor internalization in the PFC neurons were dependent on the direction of PHM (Figures S6), supporting the importance of the direction of accelerations.

The roles of FSS in organismal homeostasis have been extensively documented for vascular endothelial cells with particular reference to its regularity and magnitude (Galie et al., 2015, Kadohama et al., 2007). In contrast, positive aspects of mechanical regulation of brain functions have been poorly documented, although nervous cells and systems can be mechanically modulated during physiological processes including normal development (Karkali and Martin-Blanco, 2017) and sleep induction (Kompotis et al., 2019). To date, mechanical stress on the head has mainly been implicated in damaging outcomes such as traumatic brain injury (Levy Nogueira et al., 2016). However, given the recent studies describing relatively “sparse” distribution of nervous cells in the brain (Murakami et al., 2018) as well as the importance of interstitium in organismal functions, it is reasonable to hypothesize that interstitial space plays significant regulatory roles in various brain-related capabilities and potentials.

The exercise (treadmill running)- or PHM-dependent desensitization of PFC neurons to 5-HT that we observed in this study may not simply represent the down-regulation of 5-HT_2A_ receptor signaling, but may contribute to the homeostasis in the brain. Exercise is known to increase 5-HT production and release in rodent brain (Chaouloff, 1989, Meeusen and De Meirleir, 1995). However, such effects of exercise are site and time dependent, as 5-HT concentration in rodent cerebral cortex stays unchanged (Blomstrand et al., 1989) or even decreases after exercise (Gomez-Merino et al., 2001). Therefore, 5-HT_2A_ receptor internalization observed after a week of daily treadmill running (Figures 1I, 1J, S3D, and S3E) is likely to be instigated ligand independently, rather than resulting from increased local 5-HT concentration in the PFC. In addition, extracellular 5-HT concentration in rat brain has been reported to be significantly decreased by isoflurane anesthesia (Mukaida et al., 2007), which was used during our PHM experiments. Ligand-dependent internalization of 5-HT_2A_ receptor (Schmid et al., 2008) thus appeared unlikely to be responsible for the PHM-induced suppression of HTR.

We demonstrate that PKCγ is involved in FSS-induced 5-HT_2A_ receptor internalization in neuronal cells *in vitro* and inhibition of PKC eliminates the effects of PHM on PFC neurons *in vivo* (Figure 4 and S7). PKCγ, a member of conventional PKC (cPKC) family, is exclusively expressed post-synaptically in neurons of the central nervous system and is involved in several neuronal functions, including long-term potentiation and long-term depression (Hashimoto et al., 1988, Saito and Shirai, 2002). However, the role of PKCγ in exercise effects on brain functions has not been distinctly documented to date, although it has been reported that long-term exposure to a running wheel remarkably increased PKCγ expression and activity in hippocampal and cortical tissues of adult mice (Rao et al., 2015). Other cPKC family members are known to be involved in cellular responses to mechanical stresses (Traub and Berk, 1998), whereas mechanical regulation of PKCγ has not been demonstrated thus far. We speculate that such lack of studies on mechanical role of PKCγ may be partly due to the absence of notion that physiological functions of brain can be mechanically regulated.

Biocompatible hydrogel introduction in mouse PFCs eliminated the suppressive effect of PHM on HTR (Figure 5). Gelation of interstitial fluid may have three possible mechanisms damaging the surrounding tissue: (1) mechanical stress caused by high elastic modulus, (2) compression of surrounding tissue by swelling pressure, and (3) chemical stimulus by functional groups. To minimize the possibilities of these undesired consequences, the polymer concentration of pre-gel solutions was lowered to 25 g/L, which was confirmed to have negligible adverse effect on nerves in rabbit eyes (Hayashi et al., 2017). In our study, hydrogel introduction neither delayed HTR after 5-HTP administration (Figure S8D) nor altered 5-HT_2A_ receptor expression in the PFC (Figures S8E and S8F). Furthermore, neuronal survival or apoptosis was unaltered in hydrogel-introduced mouse PFCs (Figures S8G and S8H). Collectively, the loss of suppressive effect of PHM on HTR (Figures 5B and 5C) is likely to result from hydrogel-mediated alteration in interstitial fluid dynamics. Still, there may be unspecified effects of hydrogel introduction on the PFC neurons, particularly given that the hydrogel may alter the stiffness of extracellular matrix and the elasticity of the brain, which is known to affect the neurological physiology, pathology, and development (Li et al., 2017).

We expect that the effect of mechanical forces is not only limited to 5-HT_2A_ receptor signaling in the PFC but also relates to other cellular and molecular events involved in normal or healthy brain functions and conditions. Yet, the strict specificity of HTR for 5-HT_2A_ receptor signaling intensity in rodents' PFC neurons (Gonzalez-Maeso et al., 2007) enabled us to specifically dissect the effects of exercise (treadmill running) or PHM from complex brain functions. Our imaging-based analysis of the interstitial fluid dynamics combined with our simulative calculation indicates that intracerebral interstitial cells are subjected to FSS with an average magnitude of a few pascals, which coincides with the shear stress that protects vascular endothelial cells from inflammatory reactions (Hahn and Schwartz, 2009) or renders osteocytes mechanosensory cells in the context of mechanical loading-dependent bone homeostasis (Tatsumi et al., 2007, Weinbaum et al., 1994). Given the similarities between nervous and other types of cells with regard to intracellular signal activation by mechanical stretching (Lindqvist et al., 2010, Oldenhof et al., 2002, Sawada et al., 2001), mechano-responsive machinery may be commonly shared by a variety of different types of cells. We speculate that FSS with magnitude of a few pascals might be universally involved in organismal homeostasis. The significant contribution of cerebrospinal fluid flow to the brain homeostasis has been shown particularly in the system for waste clearance called the *glymphatic pathway’* (Iliff et al., 2012), whereas our study sheds light on another facet of the importance of extravascular fluid movement in the brain.

In summary, we have shown that mechanical perturbation on the head can modulate physiological brain functions of rodents. Further studies on the nervous system from mechanobiological perspectives will provide cues to unforeseen approaches to clinical problems related to brain malfunction and contribute to development of novel simple, safe, inexpensive, and broadly applicable therapeutic/preventative procedures for human brain diseases and disorders.

### Limitations of the Study

There are several limitations of this study. We were unable to use mice in some of the animal experiments carried out in this project. Because of the issue on body size of mice, we needed to use rats to analyze physical matters and factors related to our study, including the measurement of accelerations at the heads. Although HTR is observed both in mice and rats (Bedard and Pycock, 1977, Goodwin and Green, 1985), we cannot thoroughly exclude the possibility that there are some differences in the mechanical regulations of 5-HT_2A_ receptor signaling in their PFC neurons.

We were also unable to test the response of primary neurons, which were prepared from mouse cerebral cortex or hippocampus, to FSS of reasonable magnitudes because of their easy detachment from the substrates by FSS. However, FSS is known to activate PKC (Kroll et al., 1993, Traub and Berk, 1998) and PKC-dependent GPCR internalization is observed in various types of cells (Chapell et al., 1998, Liles et al., 1986, Park et al., 2009). Furthermore, many of the cellular responses to mechanical forces lack strict cell specificity (Iskratsch et al., 2014). Taken together, we anticipate that 5-HT_2A_ receptor internalization represents a physiological response of neurons to FSS.

Although electrical stimulation was turned on only once or twice during the first 5 min of the 30-min treadmill running on the first day of the 1-week treadmill running period as mentioned previously, we cannot entirely preclude the possible effects of mental stress concomitant with forced treadmill running. In addition, considering possible unspecified influences of hydrogel on mouse PFC, elimination of the effects of PHM by hydrogel introduction (Figure 5) may not entirely confirm or solidify the role of intracerebral interstitial fluid flow in PHM-induced HTR suppression and 5-HT_2A_ receptor internalization. Further studies are required to address these issues.

## Methods

All methods can be found in the accompanying Transparent Methods supplemental file.

## Data and Code Availability

All relevant data are available from the corresponding author upon reasonable request.

## Supplemental Information

Document S1. Transparent Methods, Figures S1–S8, and Table S1
