# Supplementary material for: Mechanical Regulation Underlies Effects of Exercise on Serotonin-Induced Signaling in the Prefrontal Cortex Neurons
Source: iScience. 2020 Jan 31;23(2):100874. doi: 10.1016/j.isci.2020.100874 (PMC7016263; doi:10.1016/j.isci.2020.100874)
Supplement: Document S1. Transparent Methods, Figures S1–S8, and Table S1 [file mmc1.pdf]

iScience, Volume *23*

Supplemental Information

Mechanical Regulation Underlies Effects of Exercise on Serotonin-Induced Signaling in the Prefrontal Cortex Neurons

Youngjae Ryu, Takahiro Maekawa, Daisuke Yoshino, Naoyoshi Sakitani, Atsushi Takashima, Takenobu Inoue, Jun Suzurikawa, Jun Toyohara, Tetsuro Tago, Michiru Makuuchi, Naoki Fujita, Keisuke Sawada, Shuhei Murase, Masashi Watanave, Hirokazu Hirai, Takamasa Sakai, Yuki Yoshikawa, Toru Ogata, Masahiro Shinohara, Motoshi Nagao, and Yasuhiro Sawada

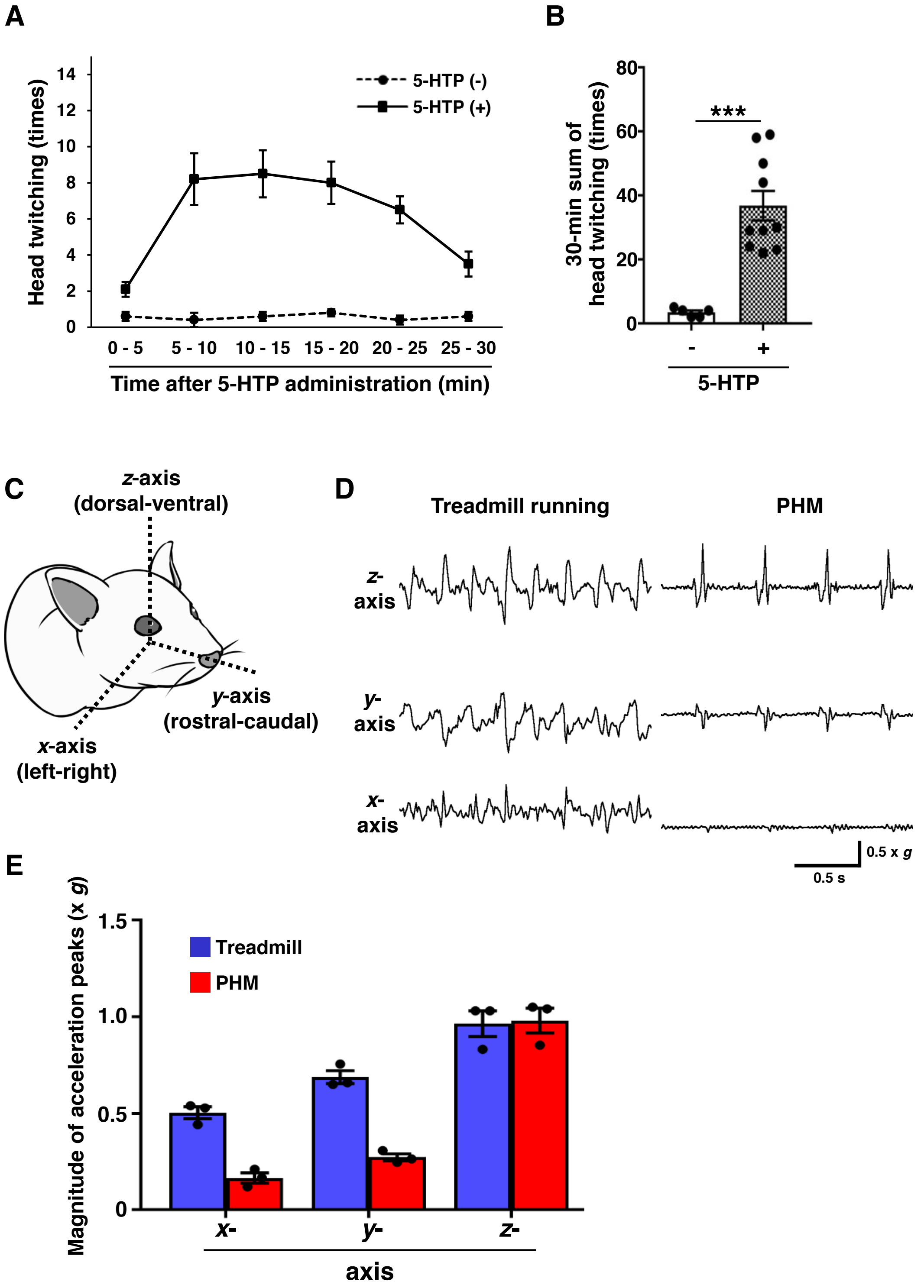

Figure S1. Counting Head Twitching after 5-HTP Administration and Measurement of Accelerations Generated at Rats' Heads during Treadmill Running and PHM, Related to Figure 1

(A and B) Quantification of 5-HTP-induced HTR. Line chart of head twitching count in 5-min blocks for 30 min (A) and histogram of total head twitching count (B) after administration of 5-HTP (5-HTP; n = 10) or saline (Control; n = 5). $^{***}p < 0.001$ (unpaired t test).

(C) Definition of *x*-(left-right), *y*-(rostral-caudal), and *z*-(dorsal-ventral) axes used in this study.

(D) Accelerations generated at the rats' heads during treadmill running (velocity: 20 m/min) and PHM (frequency: 2 Hz). The head drop by the PHM system was adjusted to 5 mm to produce 1.0 x *g* vertical acceleration peaks. Right-angled scale bar, 0.5 x *g* / 0.5 s. Images are representative of three independent experiments with similar results.

(E) Peak magnitudes of accelerations for *x*-, *y*-, and *z*-axes during treadmill running and PHM (n = 3 rats for each group).

Data are represented as means ± SEM.

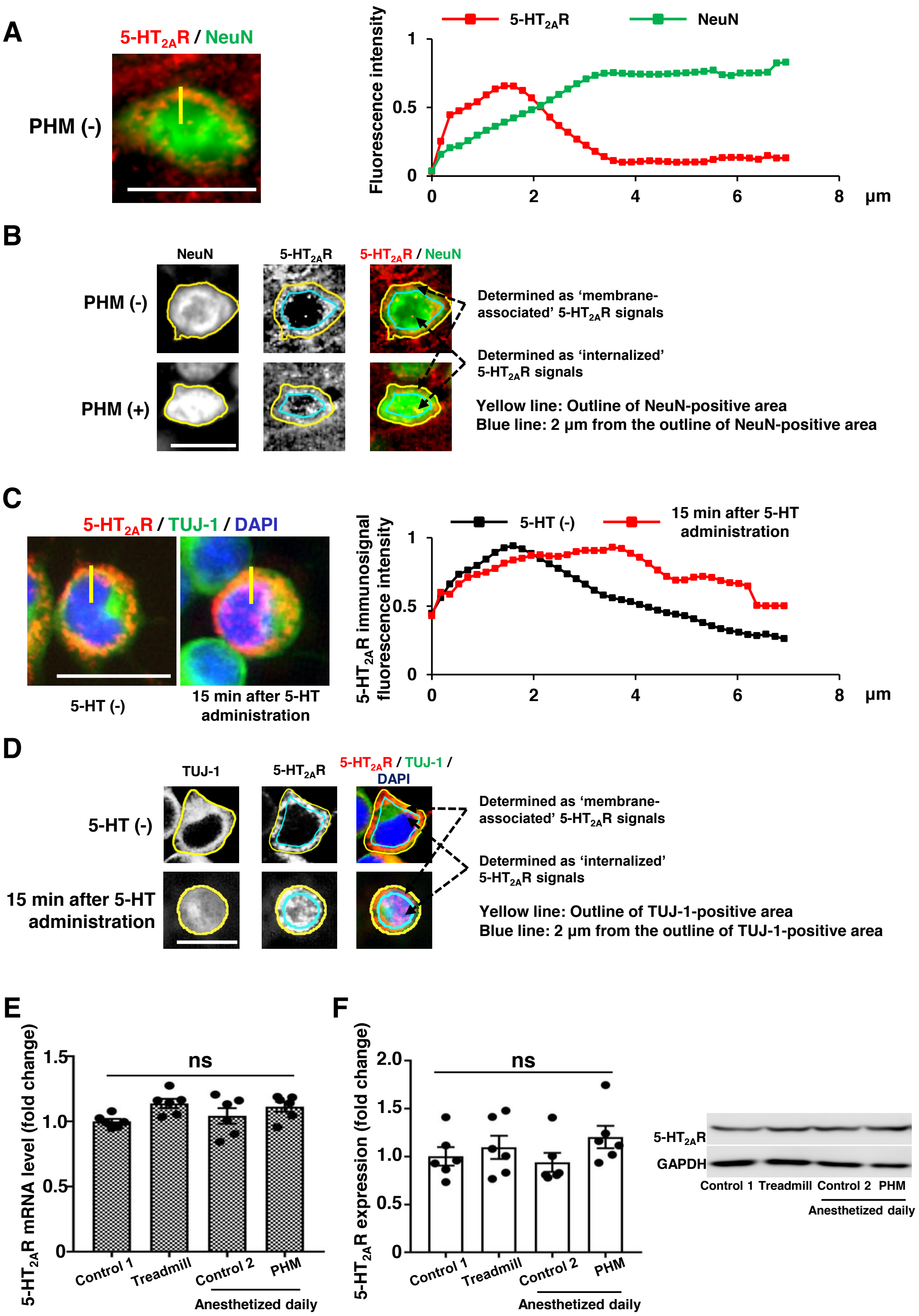

Figure S2. Representation of 5-HT$_{2A}$ Receptor Internalization in Mouse PFC Neurons and Neuro2A Cells, Related to Figures 1, 3, 4, 5, S3, S4, S6, and S7

(A) Anti-5-HT_{2A} receptor immunosignals along the soma margins of mouse PFC neurons were predominantly distributed within 2 μm from their outlines. Intensities of anti-5-HT_{2A} receptor (5-HT_{2A}R; red) and anti-NeuN (green) immunosignals were quantified along the line perpendicular to the soma outlines defined by anti-NeuN immunosignals. Scale bar, 20 μm (left). Relative signal intensities were scaled with the highest intensity in the individual cells set at 1. The graphed lines represent the mean values from 10 PFC neurons with apparent marginal distribution of anti-5-HT_{2A} receptor immunosignals (right).

(B) Schematic representation of the definition as to 'membrane-associated' and 'internalized' anti-5-HT_{2A} receptor immunosignals used for the quantification in Figures 1J, 5E, S3E, S3J, S4E, S4J, S6E, S6J, and S7D. Yellow lines indicate the outlines of somas defined by anti-NeuN immunosignals. Blue lines represent 2 μm inside the soma outlines. Scale bar, 20 μm.

(C) Quantification of the distribution of anti-5-HT_{2A} receptor immunosignals in Neuro2A cells with and without 5-HT treatment. The intensity of anti-5-HT_{2A} receptor (5-HT_{2A}R; red) immunosignals were quantified along the line perpendicular to the soma outlines of Neuro2A, either treated or left untreated with 5-HT (10 μM, 15 min). Intensities of anti-5-HT_{2A} receptor immunosignals were quantified along the line perpendicular to the soma outlines defined by anti-TUJ-1 (green) immunosignals (left), and scaled as in (A). Scale bar, 20 μm. The graphed lines individually represent the mean values from 20 Neuro2A cells with and without 5-HT treatment (right).

(D) Schematic representation of the definition as to 'membrane-associated' and 'internalized' anti-5-HT_{2A} receptor (5-HT_{2A}R; red) immunosignals used for the quantification in Figures 3C and 4D. Yellow lines indicate the outlines of neuronal

somas defined by anti-TUJ-1 (green) immunosignals. Blue lines represent 2 μm inside from the soma outlines. Scale bar, 20 μm.

(E and F) Neither treadmill running nor PHM significantly alters the expression level of 5-HT_{2A} receptor in mouse PFC. mRNA (E) and protein (F) expressions of 5-HT_{2A} receptor in mouse PFC were quantified with the mean values of control 1 set as 1 ($p = 0.095$ and $p = 0.36$ for ns, one-way ANOVA with post hoc Bonferroni test; $n = 6$ mice for each group). Controls 1 and 2 were defined as in Figure 1. Data are represented as means ± SEM. ns, not significant.

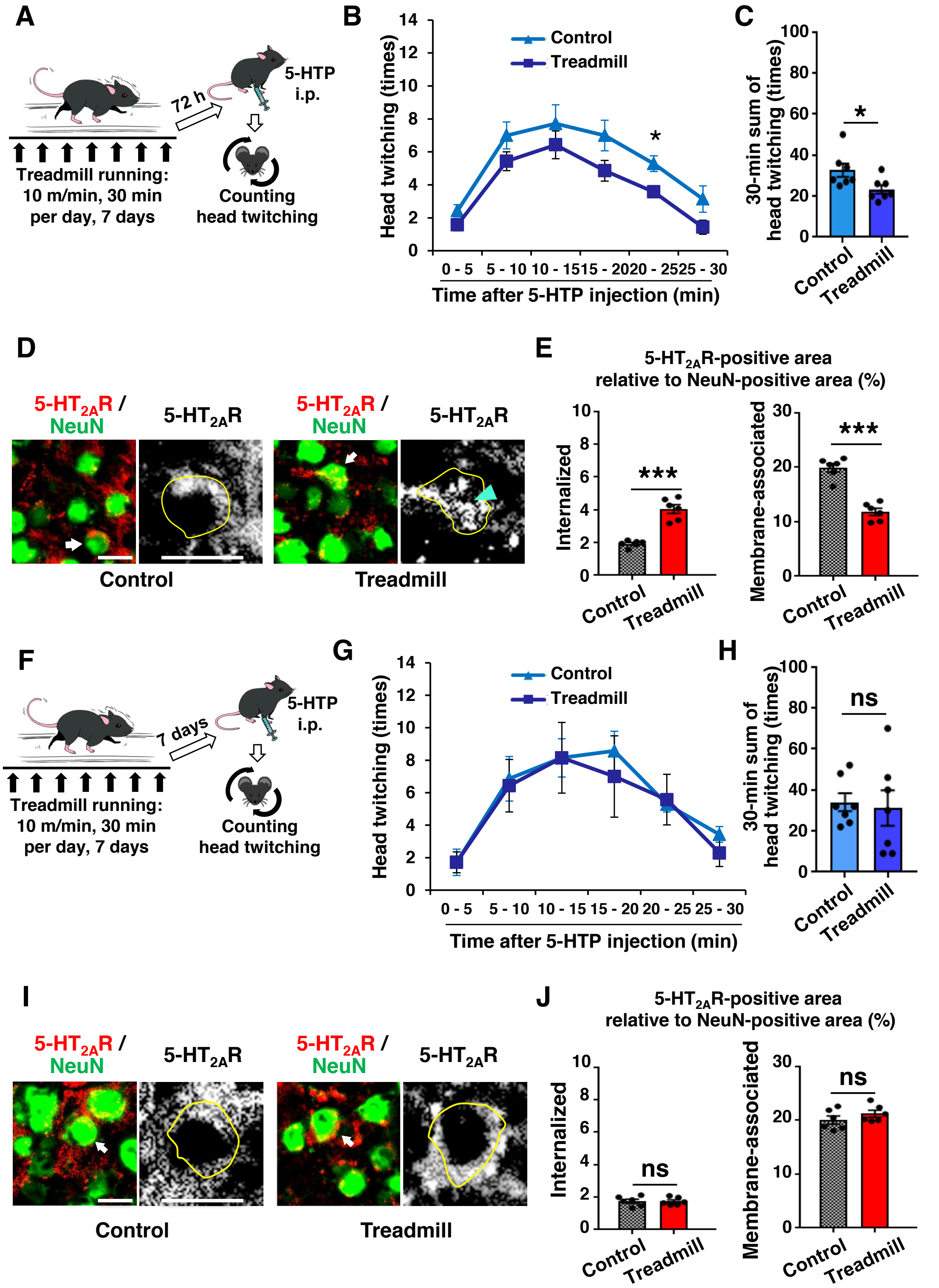

Figure S3. Longer-Term Effects of Treadmill Running on HTR and 5-HT$_{2A}$ Receptor Internalization in the PFC Neurons of Mice, Related to Figure 1

(A-E) The effects of 1-week daily treadmill running remain 72 h after its last bout. (A) Schematic representation of experimental protocol for analysis of 72-h effects of treadmill running on HTR. 5-HTP administration for HTR test was conducted 72 h after the last bout of 1-week daily treadmill running. (B and C) Count of head twitching in 5-min blocks (B) and for 30 min (C) post-5-HTP administration (C, $p = 0.033$, unpaired t test; $n = 7$ mice for each group). (D) Micrographic images of anti-5-HT_{2A} receptor (5-HT_{2A}R; red) and anti-NeuN (green) immunostaining of mouse PFC are presented as in Figure 1I. Scale bars, 20 μm. (E) Quantification of internalized and membrane-associated 5-HT_{2A} receptor-positive area relative to NeuN-positive area in mouse PFC is presented as in Figure 1J (internalized: $p < 0.001$, membrane-associated: $p < 0.001$, unpaired t test; $n = 6$ mice for each group).

(F-J) The effects of 1-week daily treadmill running do not remain 7 days after its last bout. (F) Schematic representation of experimental protocol for analysis of 7-day effects of treadmill running on HTR. 5-HTP administration for HTR test was conducted 7 days after the last bout of 1-week daily treadmill running. (G-J) HTR and 5-HT_{2A} receptor internalization in PFC neurons of mice were analyzed as in (B-E) (H, $p = 0.77$, unpaired t test; $n = 7$ mice for each group) (J, internalized: $p = 0.98$, membrane-associated: $p = 0.27$, unpaired t test; $n = 6$ mice for each group). Scale bars, 20 μm.

Controls in (B-E and G-J) represent mice that were placed on the belt of treadmill machine without turning on the treadmilling (30 min per day, 7 days). Data for the samples in (D, E, I and J) were obtained from mice infused with 4% PFA/PBS immediately after HTR test shown in (B, C, G and H). Data are represented as means ± SEM. $^{*}p < 0.05$; $^{***}p < 0.001$; ns, not significant. See also Figure S2.

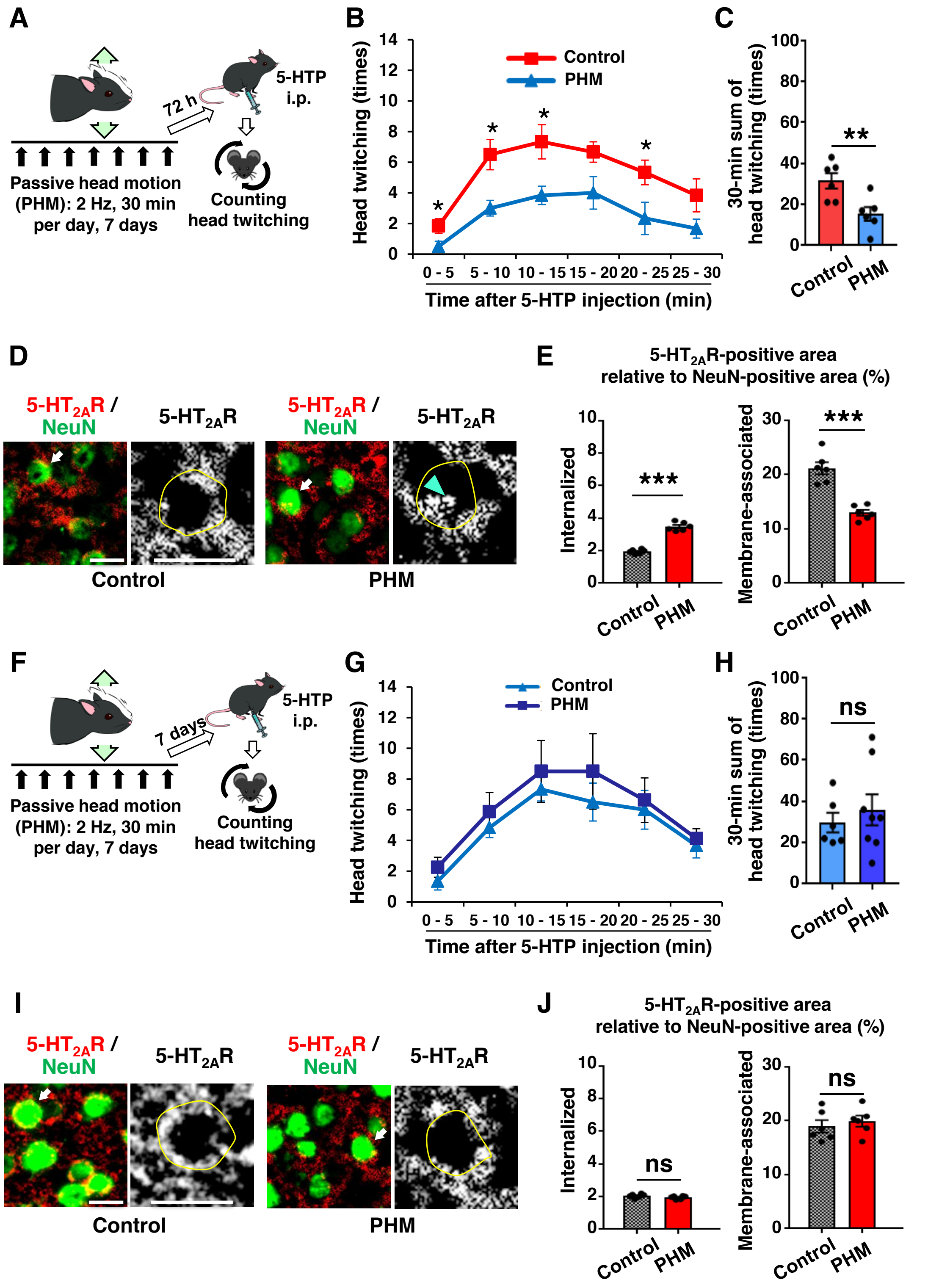

Figure S4. Longer-Term Effects of PHM on HTR and 5-HT$_{2A}$ Receptor Internalization in the PFC Neurons of Mice, Related to Figure 1

(A-E) The effects of 1-week daily PHM remain 72 h after its last bout. (A) Schematic representation of experimental protocol for analysis of 72-h effects of PHM on HTR. 5-HTP administration for HTR test was conducted 72 h after the last bout of 1-week daily PHM. (B-E) HTR and 5-HT_{2A} receptor internalization in PFC neurons of mice were analyzed as in Figures S3B-S3E (C, $p = 0.0095$, unpaired t test; $n = 6$ mice for each group) (E, internalized: $p < 0.001$, membrane-associated: $p < 0.001$, unpaired t test; $n = 6$ mice for each group). Scale bars, 20 μm.

(F-J) The effects of 1-week daily PHM do not remain 7 days after its last bout. (F) Schematic representation of experimental protocol for analysis of 7-day effects of treadmill running on HTR. 5-HTP administration for HTR test was conducted 7 days after the last bout of 1-week daily treadmill running. (G-J) HTR and 5-HT_{2A} receptor internalization in PFC neurons of mice were analyzed as in Figures S3B-S3E (H, $p = 0.53$, unpaired t test; $n = 6$ mice for control group, $n = 8$ mice for PHM group) (J, internalized: $p = 0.16$, membrane-associated: $p = 0.54$, unpaired t test; $n = 6$ mice for each group). Scale bars, 20 μm.

Controls in (B-E and G-J) represent mice that were anesthetized, and placed in a prone position with their heads on the platform that was left unoscillated (30 min per day, 7 days). Data for the samples in (D, E, I and J) were obtained from mice infused with 4% PFA/PBS immediately after HTR test shown in (B, C, G and H). Data are represented as means ± SEM. $^{*}p < 0.05$; $^{**}p < 0.01$; $^{***}p < 0.001$; ns, not significant. See also Figure S2.

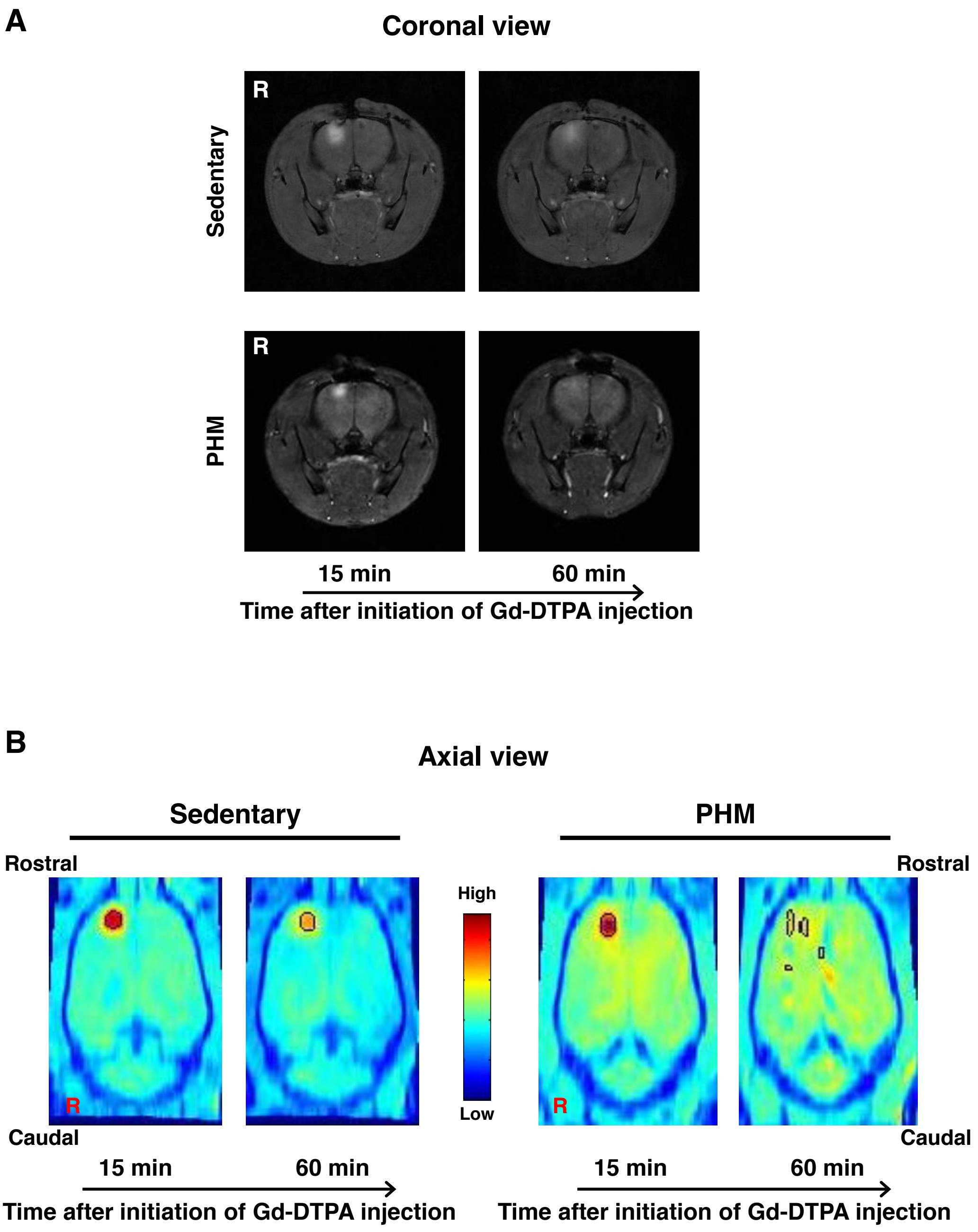

Figure S5. MRI Analysis of Spreading of Gd-DTPA Injected in Rats' PFC, Related to Figure 2

(A) Representative coronal view slices of MRI scanned before and after interventions (PHM or sedentary). 'R' indicates the right side. Each image is representative of five rats.

(B) Pseudo-color presentation of MR signal intensity. Gd-DTPA clusters were defined as voxels of top 0.05% intensity marked with black lines. The original MRI data for the slices shown in (A) and for the intensity shown in (B) are identical (from the same rats) in both sedentary and PHM-applied rats.

A

Property	Value
Pressure (ΔP; mm Hg)	0.93
Viscosity (μ; mPa·s)	1 - 20 #
Spread distance along *x*-axis (Δx; μm)	0.4074
Spread distance along *y*-axis (Δy; μm)	0.6222
Spread distance along *z*-axis (Δz; μm)	0.4370
Velocity of interstitial fluid flow along *x*-axis ($u_{\infty,x}$; μm/s)	0.8148
Velocity of interstitial fluid flow along *y*-axis ($u_{\infty,y}$; μm/s)	1.2592
Velocity of interstitial fluid flow along *z*-axis ($u_{\infty,z}$; μm/s)	0.8741

B

Fluid shear stress (τ_x) along *x*-axis at the cell surface:

$$\tau_x = \frac{\mu u_{\infty,x}}{\sqrt{K_{p,x}}}$$

$$K_{p,x} = \frac{\mu u_{\infty,x} \Delta x}{\Delta P}$$

, where $K_{p,x}$ is the Darcy permeability of brain tissue along *x*-axis.

The shear stresses along *y*- and *z*-axes can be calculated in a similar manner.

When the values listed in **A** are introduced in these equations, the magnitude of fluid shear stress is estimated as 0.86 - 3.9 Pa.

Table S1. Simulative Calculation of the Magnitude of PHM-Generated Fluid Shear Stress on the PFC Neurons, Related to Figure 2

(A) Values referenced for simulative calculation of the magnitude of fluid shear stress that PHM generated in the PFC. All referenced values except viscosity (marked with ♯) were drawn from analyses with ICP measurement and Gd-DTPA-enhanced MRI (Figures 2 and S5). The property of interstitial fluid viscosity was referenced from previous studies (Huang and Bonn, 2007; Sugiyama et al., 2013; Yao et al., 2013).

(B) Calculation of the magnitude of PHM-generated fluid shear stress. Fluid shear stress (τ) at the cell surface can be calculated as reported previously (Tarbell and Shi, 2013).

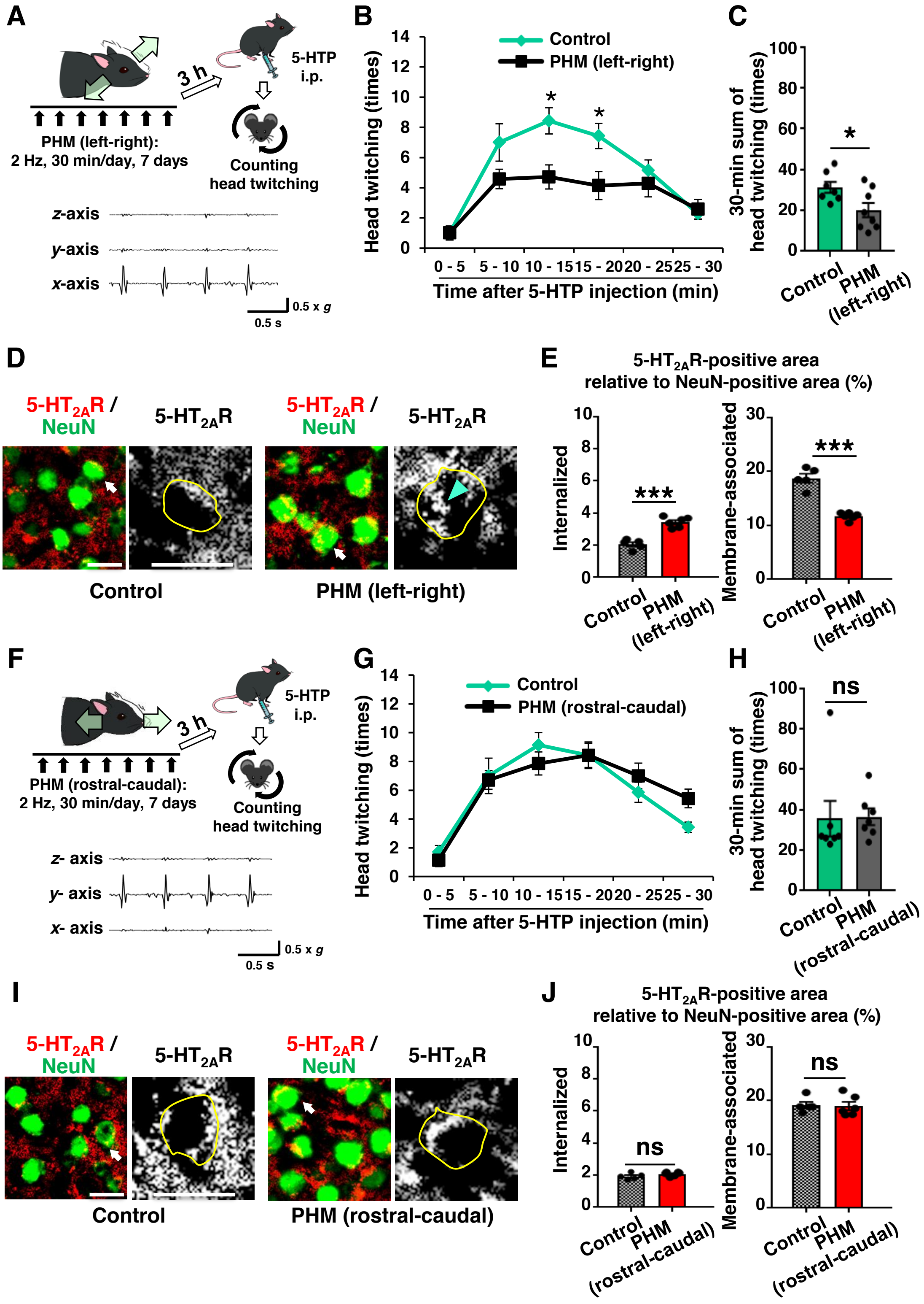

Figure S6. Effects of PHM in the Directions Other Than Vertical One, Related to Figure 2

(A-E) PHM in the left-right direction suppresses HTR and induces 5-HT_{2A} receptor internalization in PFC neurons of mice. (A, top) Schematic representation of experimental protocol for analysis of the effects of left-right PHM on HTR. 5-HTP administration for HTR test was conducted 3 h after the last bout of 1-week daily PHM. (A, bottom) The PHM system was adjusted to selectively produce 1.0 x *g* acceleration peaks along the *x*-axis. Accelerations of the rats' heads during PHM were measured as in Figure S1D, and presented following the definition of each axis shown in Figure S1C. Right-angled scale bar, 0.5 x *g* / 0.5 s. Images are representative of three independent experiments with similar results. (B-E) HTR and 5-HT_{2A} receptor internalization in PFC neurons of mice were analyzed as in Figures S3B-S3E (C, $p = 0.029$, unpaired t test; n = 7 mice for control group, n = 8 mice for PHM group) (E, internalized: $p < 0.001$, membrane-associated: $p < 0.001$, unpaired t test; n = 5 mice for control group, n = 6 mice for PHM group). Scale bars, 20 µm.

(F-J) PHM in the rostral-caudal direction neither suppresses HTR nor induces 5-HT_{2A} receptor internalization in PFC neurons of mice. (F, top) Schematic representation of experimental protocol for analysis of the effects of rostral-caudal PHM on HTR. 5-HTP administration for HTR test was conducted 3 h after the last bout of 1-week daily treadmill running. (F, bottom) The PHM system was adjusted to selectively produce 1.0 x *g* acceleration peaks along the *y*-axis. Accelerations at the rats' heads were measured and presented as in (A, bottom). (G-J) HTR and 5-HT_{2A} receptor internalization in PFC neurons of mice were analyzed as in Figures S3B-S3E (H, $p = 0.92$, unpaired t test; n = 7 mice for each group) (J, internalized: $p = 0.16$, membrane-associated: $p = 0.88$, unpaired t test; n = 5 mice for control group, n = 6 mice for PHM group). Scale bars, 20 µm.

Controls in (B-E and G-J) represent mice that were anesthetized, and placed in a prone position with their heads on the platform that was left static (30 min per day, 7 days). Data for the samples in (D, E, I and J) were obtained from mice infused with 4% PFA/PBS immediately after HTR test shown in (B, C, G and H). Data are represented as means ± SEM. $^{*}p < 0.05$; $^{***}p < 0.001$; ns, not significant. See also Figure S2.

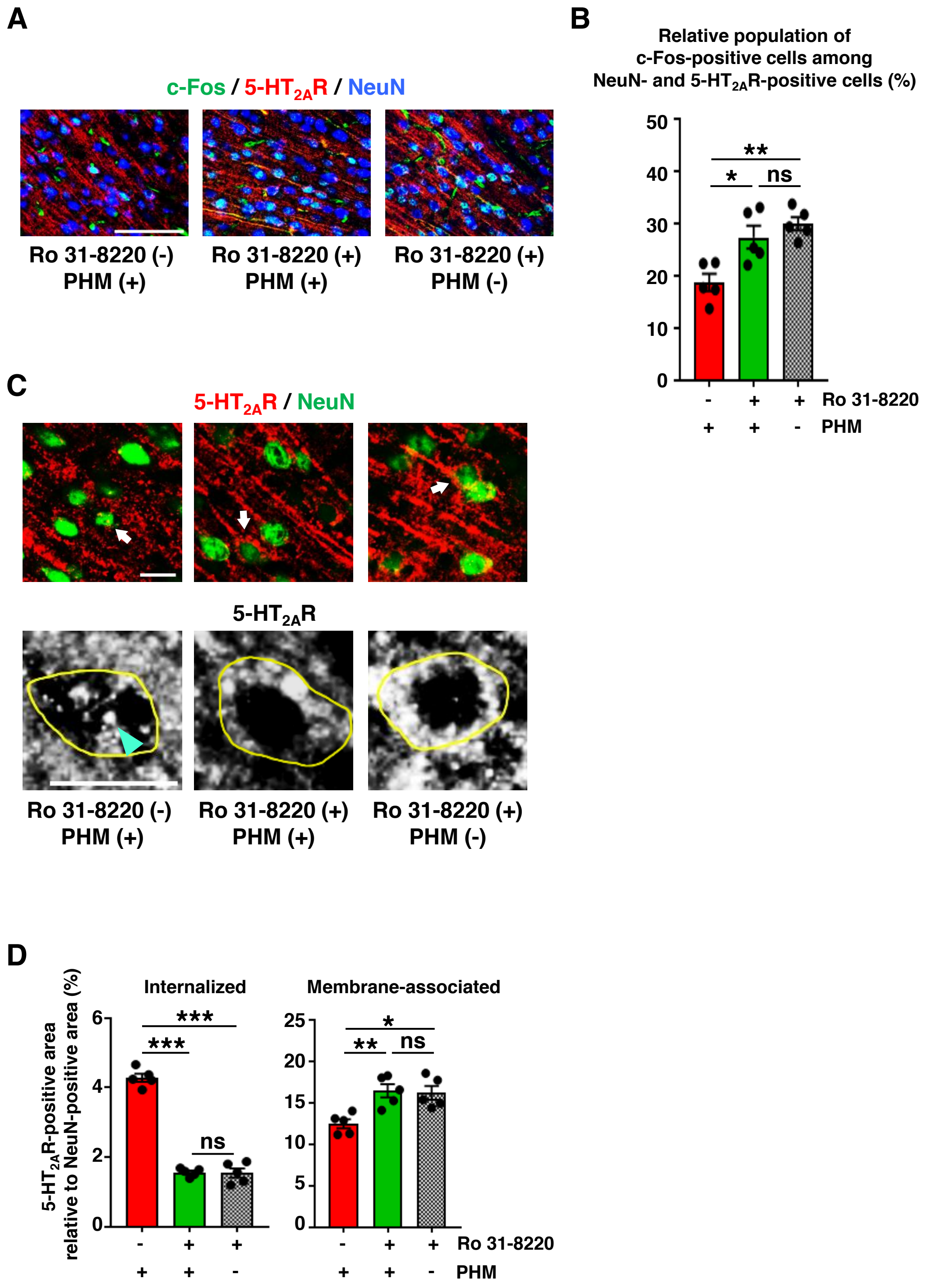

Figure S7. PKC Inhibition Eliminates the Effect of PHM on c-Fos Expression and 5-HT_{2A} Receptor Internalization in Mouse PFC Neurons, Related to Figure 4

(A) Representative micrographic images of anti-c-Fos (green), anti-5-HT_{2A} receptor (red) and anti-NeuN (blue) immunostaining of the PFC of mice administered with Ro 31-8220 or its vehicle (saline) prior to each bout of daily PHM. Scale bar, 100 μm. Images are representative of five mice.

(B) PKC inhibition nullified the effect of PHM on c-Fos expression in 5-HT_{2A} receptor-positive neurons of the PFC of mice administered with 5-HTP. Relative population (%) of c-Fos-positive cells out of 300 NeuN- and 5-HT_{2A} receptor-positive cells is shown (column 1 vs. 2: $p = 0.013$, column 1 vs. 3: $p = 0.0020$, column 2 vs. 3: $p > 0.5$, one-way ANOVA with post hoc Bonferroni test; n = 5 mice for each group).

(C) Representative micrographic images of anti-5-HT_{2A} receptor (5-HT_{2A}R; red) and anti-NeuN (green) immunostaining of mouse PFC from each group. High magnification images of anti-5-HT_{2A} receptor immunostaining of arrow-pointed cells are presented with a gray scale. Yellow lines represent the margins of neuronal somas outlined by anti-NeuN immunosignals, and cyan arrowhead points to internalized anti-5-HT_{2A} receptor immunosignals. Scale bars, 20 μm. Images are representative of four to five mice.

(D) Internalized and membrane-associated 5-HT_{2A} receptor-positive areas were quantified as in Figure 1J. Thirty-five to forty NeuN-positive cells were analyzed for each mouse (left chart: $p < 0.001$ for column 1 vs. 2 and column 1 vs. 3, $p > 0.5$ for column 2 vs. 3. right chart: $p = 0.0076$ for column 1 vs. 2, $p = 0.012$ for column 1 vs. 3, $p > 0.5$ for column 2 vs. 3, one-way ANOVA with post hoc Bonferroni test; n = 5 mice for each group). Data for (A-D) were obtained from mice infused with 4% PFA/PBS immediately after HTR test shown in Figures 4F and 4G.

Data are represented as means ± SEM. $^{*}p < 0.05$, $^{**}p < 0.01$, $^{***}p < 0.001$; ns, not significant. See also Figure S2.

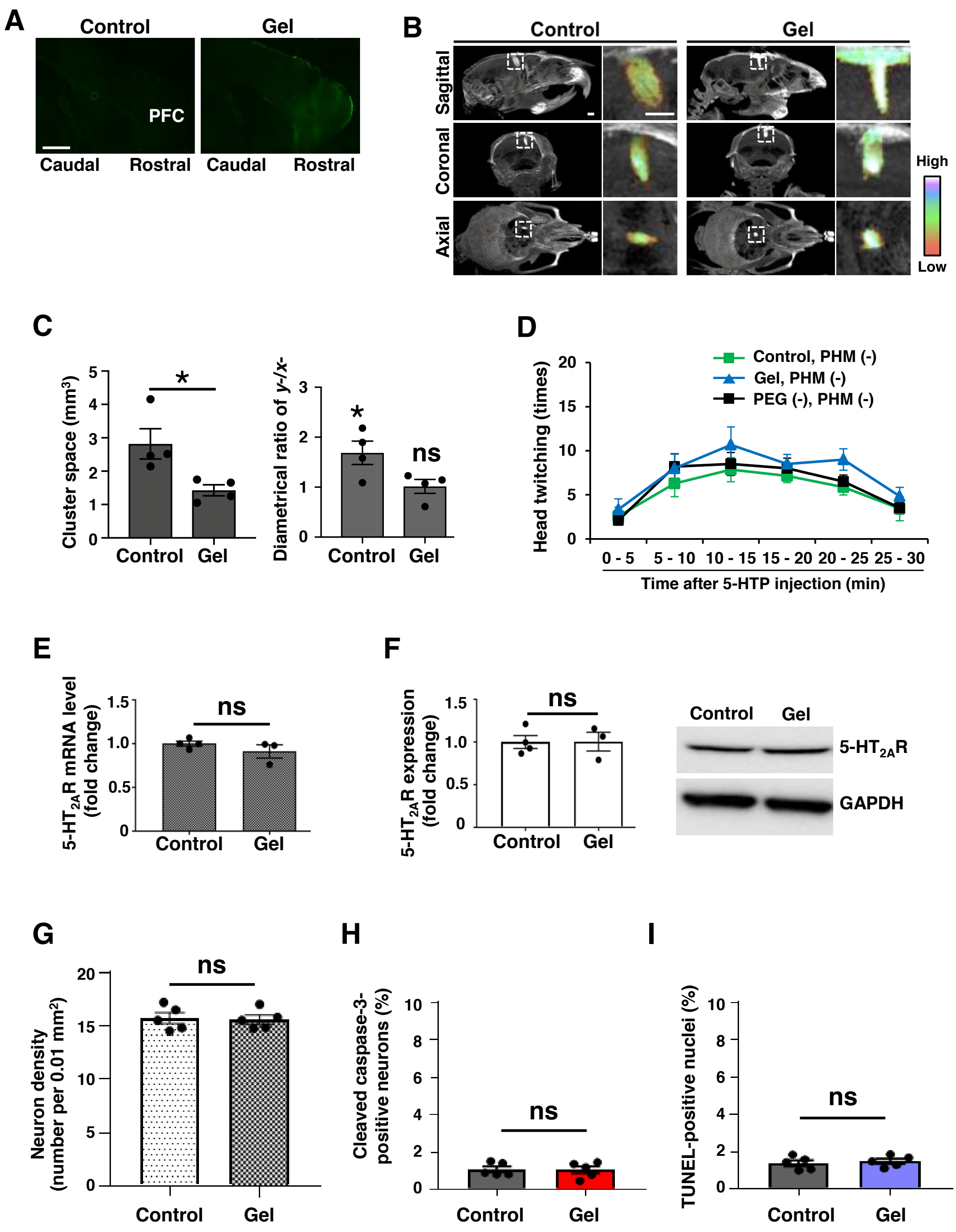

Figure S8. Hydrogel Introduction Hinders Interstitial Fluid Movement, but Does not Delay HTR after 5-HTP Injection, Alter 5-HT_{2A} Receptor Expression, or Affect Neuronal Survival/Apoptosis, Related to Figure 5

(A) Introduction of PEG hydrogel in mouse PFC. Twenty-four hours after the injection of pre-gel fluorescent PEG solution or its ungelatable control, PFC samples

were prepared. Scale bar, 1 mm. Representative sagittal images from three or four mice for each group are shown.

(B) Injected contrast medium does not enter mouse PFC after hydrogel introduction. Three hours after the injection of pre-gel PEG solution or its ungelatable control, iodine-based contrast medium (Isovist) was microinjected into the PFC. Contrast medium was visualized by μCT imaging. (B) Sagittal, coronal, and axial μCT images of Isovist-injected mouse PFC, with or without hydrogel introduction. Isovist-derived signal intensity is presented as pseudo-color in high magnification images (right) that refer to the areas indicated by dotted rectangles in low magnification images (left). Scale bars, 1 mm. Note that injected Isovist appears to remain where the needles were inserted or flow back to the brain surface without substantially entering the interstitial space of the PFC.

(C) Quantification of Isovist clusters remaining in the PFC. Isovist cluster ≥500 μm away from the brain surface were quantified for their occupying spaces (left chart, $p = 0.0278$, unpaired t test; $n = 4$ mice for each group) and diametrical ratios of *y*- (rostral-caudal) to *x*- (left-right) axis (right chart, $p = 0.026$ and 0.92 for columns 1 and 2, respectively related to the statistical hypothesis that the ratio is 1, unpaired t test; $n = 4$ mice for each group). Consistent with the anisotropic enhancement of interstitial fluid movement by PHM (Figures 2G and 2H), Isovist clusters dominantly spread along *y*-axis rather than *x*-axis in control mouse PFC (right chart).

(D) Hydrogel introduction does not delay HTR after 5-HTP administration. Data shown as ‘Control, 1st HTR’ and ‘Gel, 1st HTR’ in Figure 5B are merged with those shown as ‘Control 1’ in Figure 1B. Green and blue lines correspond to the green and blue lines in Figure 5B, respectively, and black line correspond to the black line in Figure 1B.

(E and F) Hydrogel introduction does not alter 5-HT_{2A} receptor expression in mouse PFC. Two weeks after the injection of pre-gel or its control PEG solution, mRNA (E) and protein (F) expression of 5-HT_{2A} receptor in mouse PFC were quantitatively analyzed as in Figures S2E and S2F. The mean values of control samples (column 1 or lane 1 in each panel) were set as 1 (E: $p = 0.97$, F: $p = 0.27$, unpaired t test; $n = 4$ mice for control group, $n = 3$ mice for Gel group).

(G-I) Hydrogel introduction does not affect neuronal survival/apoptosis or overall cell apoptosis. Mouse PFC sections prepared for Figure 5D were subjected to anti-NeuN and anti-cleaved caspase-3 immunostaining (G and H) or TUNEL assay (I). NeuN-positive neuronal somas were counted (G), and the ratios of NeuN- and cleaved caspase-3-double positive somas among them (H) and TUNEL-positive nuclei (I) were quantified. Each value represents an average from 5 images of 500 x 500-μm area analyzed for each mouse (G: $p = 0.85$, H: $p = 0.88$, I: $p = 0.64$, unpaired t test; $n = 5$ mice for each group).

Data are represented as means ± SEM. $^{*}p < 0.05$; ns, not significant.

Transparent Methods

Animal experiments

Animals were housed under a 12 h/12 h light/dark cycle with controlled temperature (23 - 25°C), and treated with humane care under approval from the Animal Care and Use Committee of National Rehabilitation Center for Persons with Disabilities and Tokyo Metropolitan Institute of Gerontology. Male C57BL/6 mice and Sprague-Dawley (SD) rats were purchased from Charles River (Yokohama, Japan) or SLC (Hamamatsu, Japan), acclimated to the laboratory environments for at least 1 week, randomly divided into experimental groups, and used for experiments at the age of 9 - 10 weeks (mice) and 8 - 9 weeks (rats). All the animal behavior tests were conducted between 4 pm and 8 pm. We made every effort to relieve suffers of animals during experiments and to minimize the number of animals.

Chemicals, plasmids and antibodies

All the chemicals were purchased from Sigma-Aldrich unless noted otherwise. The pcDNA 3.1-based expression vector plasmid for human 5-HT_{2A} receptor was purchased from transOMIC Technologies (Huntsville, AL). The pcDNA3-based expression vector for mCherry was obtained from the shared resources of Mechanobiology Institute in National University of Singapore. Antibodies used in this study are described in the KEY RESOURCES TABLE.

Treadmill running, PHM and acceleration analysis

As an animal model of physical exercise, mice were subjected to compulsive running using a belt drive treadmill equipped with an electrical shock system (MK-680S, Muromachi, Tokyo, Japan). We habituated the animals of both control and running groups to the treadmill system by placing them in the machine several times without

turning on the treadmill belt during the acclimation period. The electrical stimulation was turned on only once or twice during the first 5 minutes of the 30-minute treadmill running on the first day of the 1-week treadmill running period. Thereafter, we did not need to turn on the electrical shock system to have the animals keep running, perhaps because the velocities we employed (10 m/min for mice, and 20 m/min for rats) were moderate. The control mice in treadmill running experiments were placed on the belt for a defined period of time without turning on the treadmilling.

Animals (mice or rats) were subjected to PHM in a prone position using a platform that we developed to move their heads (schematically represented in Figures 1D, 2A, 2E, 4E, 5A, S4A, S4F, S6A, and S6F). During PHM, animals were kept anesthetized with 1.2% isoflurane except for the MRI study, in which we used the combination of midazolam, butorphanol and medetomidine for anesthesia. Body temperature of tested animals was maintained using a light heater. The control mice in PHM experiments were anesthetized likewise, and placed in a prone position with their heads on the platform that was left static.

To measure the accelerations at the head during treadmill running or PHM, we fixed an accelerometer (NinjaScan-Light; Switchscience, Tokyo, Japan) on the rats' heads with a surgical tape. Acceleration-specific signals were extracted and average accelerations for 3-D (*x*-, *y*- and *z*-axes) were individually calculated from 10 serial waves using the software applications provided from the manufacture and Spike2 (CED, Cambridge, UK). The PHM system was set up to produce 1.0 x *g* vertical acceleration peaks at the head of rodents examined.

Head-twitch response (HTR) test

After administration of 5-HTP (100 mg/kg) or its vehicle (normal saline) by i.p., mice were individually placed in a Plexiglas box and videotaped for 30 min using a video

camera (HDR-CX700, Sony, Tokyo, Japan). Recorded movies were reviewed to count head twitching. 5-HTP was injected 3 h after the last bout of treadmill running or PHM.

Measurement of intracerebral pressure (ICP) at rats' PFC

ICP was measured using a blood pressure telemeter (Millar, Houston, TX) with its sensor placed in rats' PFC. During ICP measurement, respiration was monitored using a respiration sensor attached to the tested rats. Low-pass (50 Hz) filtered ICP waves were analyzed using LabChart 8 (ADInstruments, Dunedin, New Zealand) software. We observed ~0.5-Hz respiration-synchronized ICP changes with ~2.5 mm Hg magnitude (peak to peak) as well as 2-Hz PHM-specific waves with ~1 mm Hg magnitude (peak to peak) (Figures 2B-2D).

In vivo analysis of the distribution dynamics of cerebral interstitial fluid using MRI

MRI Scanning: 8-week-old male SD rats were scanned in an ICON 1.0-Tesla bench top MRI system (Bruker BioSpin, Esslingen, Germany) using a T1-weighted-fast low angle shot-navigation-highers-three-dimensional (T1-FLASH-nav-highers-3-D) sequence. Parameters for the T1-FLASH-nav-highers-3-D sequence were as follows; echo time (TE): 12.0 ms, repetition time (TR): 50.0 ms, flip angle (FA): 30°, slice thickness (SL): 0.84 mm, field of view (FOV): 35 × 35 × 27 mm, voxel: 0.137 × 0.137 × 0.84 mm^3. Each scanning took 10 min.

Intracerebral microinjection of Gd-DTPA solution: Rats were first subjected to brain pre-scanning with MRI under isoflurane anesthesia, and then to intracerebral microinjection of Gd-DTPA (Magnevist®, Bayer, Leverkusen, Germany) as previously reported with some modifications. In brief, a 31-G microsyringe (Hamilton Bonaduz AG, Bonaduz, Switzerland) was stereotaxically positioned on rats

anesthetized with 2 mg/kg of midazolam (Sandoz, Basel, Switzerland), 2.5 mg/kg of butorphanol (Meiji Seika, Tokyo, Japan) and 0.15 mg/kg of medetomidine (Kyoritsu Seiyaku, Tokyo, Japan), such that 1 μl of Gd-DTPA solution (diluted to 25 mM with normal saline) could be infused with the rate fixed at 0.2 μl/min using a microsyringe pump instrument (KD scientific, Holliston, MA). The needle placement measurements were as follows; anterior: 2.5 - 3.0 mm, lateral: 1.5 - 2.0 mm, ventral: 2.0 - 3.0 mm from the bregma. After the injection, we held the microsyringe for 5 min to avoid reflux, pulled out the needle carefully, and sutured the skin.

MRI data acquisition and post-processing: Following intraparenchymal Gd-DTPA injection, rats were subjected to two serial brain MRI scans between which PHM was either applied or left unapplied (kept sedentary) for 30 min (Figure 2E). MR images were analyzed using a software package rSPM (Gaser et al., 2012), which is a modified version of SPM12 (http://www.fil.ion.ucl.ac.uk/spm/software/spm12/) developed for human neuroimaging data analyses. The original data were converted from the DICOM format to the NIfTI format. Each individual rat brain image was realigned to the first volume and spatially normalized to a template brain image provided by rSPM. The normalized data had an 86 x 121 x 66 matrix with a 0.2 x 0.2 x 0.2 mm^3 isotropic voxel. The Gd-DTPA cluster was defined as voxels with top 0.05% signal intensity in each volume.

Simulative calculation of the magnitude of fluid shear stress on the PFC neurons during PHM

Interstitial fluid flow in the brain tissue follows the Henry Darcy's law, which defines the flux density of penetrating fluid per unite time. The velocity of interstitial fluid flow (u) is assumed to approach the superficial velocity (u_∞) and zero (u = 0) at the cell surface (i.e. a no-slip condition). Using these two boundary conditions together

with the Brinkman equation, fluid shear stress (τ) at the interstitial cell surface can be obtained as described in Table S1.

Neuronal cell culture and plasmid transfection

Mouse neuroblastoma-derived Neuro2A cells (provided from Dr. Yokota, Tokyo Medical and Dental University, Japan), which exhibit neuronal phenotypes and morphology (Goshima et al., 1993; Yun et al., 2013), were cultured in DMEM (Wako, Osaka, Japan) with 10% fetal bovine serum (FBS; GE Healthcare Life Science Marlborough, MA) at 37°C in a 5% CO_2 incubator. At approximately 70% confluence in a 10-cm culture dish (Corning Life Sciences, Corning, NY), Neuro2A cells were transfected with 10 μg of a 5-HT_{2A} receptor expression vector plasmid using lipofectamine® 3000 (Thermo Fisher Scientific, Waltham, MA) according to the manufacturer's directions. Neuro2A is not a commonly misidentified cell line maintained by ICLAC. We confirmed the expression of neuronal proteins such as TUJ-1 (neuron-specific Class III β-tubulin) and the axonal formation. The cell line was not tested for mycoplasma contamination. However, when we conducted DAPI staining, we did not observe a dotted stain around the nuclei, which is characteristic of mycoplasma contamination.

Application of FSS to neuronal cells in culture

Neuro2A cells grown in a poly-D-lysine coated 35-mm culture dish (Corning Life Sciences) were cultured in serum-free DMEM for 24 h before exposure to FSS (0.91 Pa) for 30 min. As we previously reported (Yoshino et al., 2013), a parallel plate flow-chamber and roller pump (Masterflex, Cole-Parmer, IL) were used to apply FSS. The flow-chamber, which was composed of a cell culture dish, a polycarbonate I/O unit, and a silicone gasket, generated a 23-mm-long 10-mm-wide 0.5-mm-high flow channel. The flow circuit was filled with serum-free medium. To maintain pH and

temperature of culture medium, we used a 5% CO_2-containing reservoir and a temperature-controlled bath.

Live cell imaging to monitor intracellular calcium (Ca^{2+}) concentration

We conducted live cell imaging to monitor intracellular Ca^{2+} concentration after 5-HT administration. Neuro2A cells grown in a 10-cm dish were transfected with 10 μg of 5-HT_{2A} receptor and 5 μg of mCherry expression plasmid vector using lipofectamine® 3000 (Thermo Fisher Scientific). Forty-eight hours after transfection, cells were transferred and grown overnight in a poly-D-lysine coated 35-mm dish, which was placed in a 37ºC/5%-CO_2 plate type incubator (Tokai Hit, Fujinomiya, Japan) attached to a microscope (BZ-9000 HS, Keyence, Osaka, Japan). Then, a flow chamber was set to the dish plate and FSS was applied. Cells, either left unexposed or exposed to FSS (average 0.91 Pa, 30 min), were loaded with Fluo 4-AM dye (DOJINDO, Kumamoto, Japan) for 60 min in a 37ºC/5%-CO_2 incubator. Cells were then placed on an incubator-attached fluorescence microscope, and treated with 5-HT (10 μM). Ten minutes after 5-HT administration, green fluorescence emitted from Fluo 4-AM bound to Ca^{2+} was viewed with the microscope.

Immunohistochemical analysis of mouse PFC

After the completion of HTR tests, animals were anesthetized by administrating combinations of midazolam, butorphanol and medetomidine, and perfused with 4% PFA in phosphate-buffered saline (PBS) transcardially. Then, brains were excised, post-fixed in 4% PFA in PBS for additional 24 h at 4ºC, and stored in 30% sucrose/PBS until they sank. Fixed brains were frozen in OCT (Sakura Finetek, Tokyo, Japan) and cut into 20 μm-thick sagittal sections using a cryostat (CM3050S, Leica Microsystems, Wetzlar, Germany). Sliced sections were permeabilized with 0.1% Tween-20 in Tris-buffered saline, blocked with 4% donkey serum (Merck

Millipore, Burlington, MA), stained by incubating with primary antibodies at appropriate concentrations followed by their species-matched secondary antibodies conjugated with Alexa Fluor 488, 568 or 645 (Thermo Fisher Scientific), and viewed with a fluorescence microscope (BZ-9000 HS, Keyence).

Immunocytochemical analysis of Neuro2A cells

Neuro2A cells grown in a poly-D-lysine-coated dish were washed once with ice-cold PBS, fixed with 4% PFA in PBS, permeabilized with 0.1% Triton X-100 in PBS, incubated with a blocking solution (10% FBS, Thermo Fisher Scientific), and stained using target-specific primary and their species-matched secondary antibodies. DAPI was used for nuclear staining. Cells were then viewed with a fluorescence microscope (BZ-9000 HS, Keyence).

Quantitative analysis of 5-HT$_{2A}$ receptor internalization in vivo and in vitro

To quantify 5-HT$_{2A}$ receptor internalization in PFC neurons, we defined 'internalized' and 'membrane-associated' 5-HT$_{2A}$ receptor-positive signals based on the distance from soma outlines and the connectivity with marginal regions. We outlined the somas of individual PFC neurons by tracing the outlines of NeuN-positive area (see yellow lines in Figure S2B). The anti-5-HT$_{2A}$ receptor immunosignals that appeared along the marginal outlines were mostly distributed within 2 μm of soma margins (Figure S2A). In the case of Neuro2A cells, we outlined their soma margins by anti-TUJ-1 (neuron-specific Class III β-tubulin) immunostaining, because anti-NeuN immunosignals did not clearly indicate them. Immunostaining of 5-HT$_{2A}$ receptor-expressing Neuro2A cells grown in a poly-D-lysine-coated culture dish revealed that the major portion of 5-HT$_{2A}$ receptor distribution was along the soma margins with its peak <2 μm away from their outlines (Figure S2C). In contrast, treatment of Neuro2A cells with 5-HT (10 μM, 15 min), which is known to induce 5-HT receptor

internalization (Baldys and Raymond, 2011; Bhattacharyya et al., 2002), altered the distributional profile of the anti-5-HT_{2A} receptor immunosignal intensity, shifting its peak >2 μm away from soma outlines (Figure S2C). Based on these observations, we defined the anti-5-HT_{2A} receptor immunosignals within 2 μm of soma outlines as 'membrane-associated' (Figures S2B and S2D). Resultantly, the anti-5-HT_{2A} receptor immunosignals distributed at >2 μm from soma outlines were defined as 'internalized' (Figures S2B and S2D). In the case of in vivo analysis (i.e. immunostaining of mouse PFC), anti-5-HT_{2A} receptor immunosignals connected with perimarginal regions were defines as 'membrane-associated', regardless of the distance from soma outlines. To avoid the possible underestimation of membrane-associated area for images in which 'peripheral' parts of cells were scanned, somas larger than 10 μm in short diameter were subjected to the quantification of 5-HT_{2A} receptor internalization. Images were analyzed using Photoshop CC (Adobe Systems, San Jose, CA) and ImageJ (NIH, Bethesda, MD) software.

We quantified 5-HT_{2A} receptor-internalization based on the ratio of internalized or membrane-associated anti-5-HT_{2A} receptor immunosignal-positive area to total soma area defined by outlining anti-NeuN (mouse PFC neurons) or anti-TUJ-1 (Neuro2A cells) immunosignal-positive area (Figures 1J, 3C, 4D, 5E, S3E, S3J, S4E, S4J, S6E, S6J, and S7D). Images were analyzed using BZ-H1C (BZ-9000 HS related application, Keyence), Photoshop CC and ImageJ software.

Quantitative PCR analysis (reverse transcription and real-time PCR)

Five hundred nanograms of total RNA extracted from mouse PFC excised immediately after cervical dislocation were subjected to reverse transcription, using ISOGEN II (NIPPON GENE, Tokyo, Japan) and PrimeScript® RT reagent Kit (TaKaRa, Kusatsu, Japan). The resulting cDNA was subjected to real-time PCR

analysis using glyceraldehyde-3-phosphate dehydrogenase (GAPDH) as an internal control in Applied Biosystems 7500 Real Time PCR System with Power SYBR Green PCR Master Mix (Thermo Fisher Scientific).

The primers (sense and antisense, respectively) were as follows: mouse *Htr2a*, 5'-TGCCGTCTGGATTTACCTGG-3' and 5’-TGGTTCTGGAGTTGAAGCGG-3’, mouse *Gapdh*, 5'-GCAAAGTGGAGATTGTTGCCAT-3' and 5'- CCTTGACTGTGCCGTTGAATTT -3'.

Immunoblot analysis

Mouse PFC tissue was excised immediately after cervical dislocation, mechanically homogenized, solubilized with sodium dodecyl sulfate (SDS) sample buffer (50 mM Tris-HCl pH 7.0, 18% glycerol, 2% SDS), and subjected to SDS-PAGE followed by anti-5-HT_{2A} receptor and anti-GADPH immunoblotting. Specific signals were visualized and quantified using Odyssey infrared imaging system (LI-COR Biosciences, Lincoln, NE) and ImageJ software.

Hindrance of interstitial fluid movement (flow) by introduction of hydrogel in mouse PFC

Preparation of a pre-mixed solution of polyethylene glycol (PEG) with functional groups: Equal volume of tetra-armed thiol-terminal (TetraPEG-SH) (Yuka-Sangyo, Tokyo, Japan) and acrylate-terminal (Tetra-PEG-ACR) (JenKem Technology, Plano, TX) PEG solutions (25 g/L in PBS) were mixed just before use (Hayashi et al., 2017). Tetra-armed polyethylene glycol without functional groups (25 g/L in PBS) was used as an ungelatable control. For the analysis of hydrogel distribution in the mouse PFC, we used Tetra-PEG-SH fluorescently labeled with a thiol-reactive probe (Merck KGaA, Darmstadt, Germany).

Intracerebral microinjection of PEG solution: A 31-G syringe (Terumo Corporation, Tokyo, Japan) was stereotaxically positioned on mice anesthetized with intraperitoneal injection of a mixture of three anesthetic agents (midazolam, butorphanol, medetomidine), such that 4 μl of pre-mixed PEG solution could be injected with the rate fixed at 0.2 μl/min using a microsyringe pump. The needle placement measurements were as follows; anterior: 1.5 - 2.0 mm, lateral: 1.5 - 2.0 mm, ventral: 1.0 - 1.5 mm from the bregma. After the injection, we held the syringe for 5 min to avoid reflux, pulled out the needle carefully, and sutured the skin.

To dissect the effects of PHM on HTR from those resulting from these experimental procedures, we gave 1-week recovery time before the first HTR test and subsequent PHM application to the mice (daily 30 min, 7 days). Three hours after the last bout of PHM, the second HTR test was conducted (see Figure 5A). Immediately following 30-min counting of head twitching after 5-HTP administration, mice were sacrificed by transcardial infusion of paraformaldehyde and subjected to histological analysis.

μCT analysis of the effect of hydrogel introduction in mouse PFC

Three hours after the injection of either the pre-gel PEG solution or its ungelatable control, 5 μl of an iodine-based contrast medium (Isovist® inj. 300, Bayer) was microinjected and visualized using μCT (inspeXio SMX-100CT, Shimadzu, Kyoto, Japan). Although contrast medium injection was conducted separately from PEG injection (i.e., the needle was pulled out and the skin was sutured once PEG infusion was completed), the stereotaxic needle placement position was identical between PEG and contrast medium injections. The rate of contrast medium infusion was fixed at 0.5 μl/min. After the injection, we held the microsyringe for 5 min to avoid reflux, pulled out the needle carefully, and sutured the skin. μCT images were obtained with

following parameters; voxel size 50 μm, 60 KeV, 58 μA, field of view 25.6 mm, matrix size 512 x 512. 3D reconstructed objects were visualized and analyzed on software for 3D morphometry (TRI/3D-BON-FCS64, RATOC System, Tokyo, Japan). The contrast medium cluster in mouse PFC was defined as voxels with ≥1.3 times signal intensity as compared to that of air.

Terminal deoxynucleotidyl transferase-mediated dUTP nick-end labeling (TUNEL) assay

Mouse PFC sections were stained using a TUNEL kit (Biotium, Fremont, CA) according to the manufacturer's protocols, counterstained with DAPI, and then viewed using a 20x objective with a fluorescence microscope (BZ-9000 HS, Keyence). The nuclei of apoptotic cells were determined by the existence of green fluorescent patches, and cell apoptosis was quantified by referring their counts to total nuclear numbers defined by DAPI staining.

Statistical analysis

All the quantitative data are presented as means ± SEM. Parametric statistical analyses were conducted by two-tailed t test for two-group comparison and ANOVA with post hoc (Bonferroni or Dunnett) for multiple (≥ 3) group comparison, using SPSS (Version 24, IBM SPSS, Chicago, IL) and PRISM (version 7, GraphPad Software, San Diego, CA). Differences were considered as significant at p values below 0.05.

Adequate sample sizes of experiments were estimated based on our pilot experiments and experience with similar measurements to ensure adequate power to detect a specified effect size. For animal behavioral analyses, at least 4 animals were assigned to each experimental group according to standard scientific conventions. We tried to reach a conclusion for each individual experiment using the smallest sample

size possible. No data were excluded except for MRI analysis, from which data from a rat with unsuccessful microinjection of Gd-DTPA were eliminated. The investigators were not blinded to any group allocations for in vivo or in vitro experiments.

CellPress

KEY RESOURCES TABLE

REAGENT or RESOURCE	SOURCE	IDENTIFIER
Antibodies		
Mouse monoclonal anti-NeuN (clone A60)	EMD Millipore (Merck)	RRID:AB_2298772 Catalog# MAB377
Rabbit monoclonal anti-phospho-p44/42 MAPK	Cell Signaling Technology	RRID:AB_2315112 Catalog# 4370
Rabbit monoclonal anti-GAPDH	Cell Signaling Technology	RRID:AB_10622025 Catalog# 5174
Mouse monoclonal anti-β-III tubulin	Abcam	RRID:AB_2256751 Catalog# ab78078
Rabbit polyclonal anti-β-III tubulin	Abcam	RRID:AB_444319 Catalog# ab18207
Rabbit polyclonal anti-phospho-MARCKS	Sigma-Aldrich	Catalog# P0370
Mouse monoclonal anti-5-HT_{2A} receptor	Santa Cruz Biotechnology	Catalog# sc-166775
Goat polyclonal anti-5-HT_{2A} receptor	Santa Cruz Biotechnology	Catalog# sc-15073
Rabbit polyclonal anti-c-Fos	Santa Cruz Biotechnology	RRID:AB_2106783 Catalog# sc-52
Rabbit polyclonal anti-PKCγ	Santa Cruz Biotechnology	RRID:AB_632234 Catalog# sc-211
Rabbit polyclonal anti-cleaved Caspase-3 (Asp175)	Cell Signaling Technology	Catalog# 9661L
Bacterial and Virus Strains		
N/A		
Biological Samples		
N/A		
Chemicals, Peptides, and Recombinant Proteins		
Lipofectamine 3000	Thermo Fisher Scientific	Catalog# L300008
Tetra-PEG-SH	Yuka-Sangyo	SUNBRIGHT PTE-200SH
Tetra-PEG-ACR	JenKem Technology	4ARM-ACLT-20K
Tetra-PEG-SH fluorescently labeled with a thiol-reactive probe	Merck KGaA	Catalog# 595504
4ARM-PEG (ungelatable PEG solution)	JenKem Technology	MW 20K
5-hydroxytryptophan (5-HTP)	Sigma-Aldrich	Catalog# H9772
Serotonin (5-HT)	Sigma-Aldrich	Catalog# H9523
Ro 31-8220 (PKC inhibitor)	Sigma-Aldrich	Catalog# R136
Magnevist (contrast agent for MRI)	Bayer	Catalog# 341108168
Isovist (contrast agent for CT)	Bayer	Catalog# 877219
Critical Commercial Assays		
PrimeScript RT reagent Kit (reverse transcription kit)	TaKaRa	Catalog# RR037A
ISOGEN II (reagent for RNA extraction)	NIPPON GENE	Catalog# 311-07361
SYBR Green Master Mix (reagent mix for qPCR)	Thermo Fisher Scientific	Catalog# 4368706
Fluo 4-AM (fluorescent Ca^{2+} probe)	DOJINDO	Product code F311
TUNEL Assay Apoptosis Detection Kit	Biotium	Catalog# 30074
Deposited Data		

N/A		
Experimental Models: Cell Lines		
Neuro2A cells	Mouse neuroblastoma	N/A
Experimental Models: Organisms/Strains		
N/A		
Oligonucleotides		
Mouse HT_{2A} receptor: 5'-TGCCGTCTGGATTTACCTGG-3' and 5'-TGGTTCTGGAGTTGAAGCGG-3'		
Mouse GAPDH: 5'-GCAAAGTGGAGATTGTTGCCAT-3' and 5'-CCTTGACTGTGCCGTTGAATTT-3'		
Recombinant DNA		
Expression vector for mCherry (pcDNA3-mCherry)	National University of Singapore.	N/A
Expression vector for human 5-HT_{2A} receptor (pcDNA3.1-5-HT_{2A} receptor)	transOMIC Technologies	N/A
Software and Algorithms		
TRI/3D-BON, TRI/3D-BON-C	RATOC Systems	N/A
ImageJ	NIH	N/A
SPSS	IBM SPSS	Version 24
PRISM	GraphPad Software	Version 7
Other		

Supplemental References

Baldys, A., and Raymond, J.R. (2011). Role of c-Cbl carboxyl terminus in serotonin 5-HT_{2A} receptor recycling and resensitization. J. Biol. Chem. *286*, 24656-24665.

Gaser, C., Schmidt, S., Metzler, M., Herrmann, K.H., Krumbein, I., Reichenbach, J.R., and Witte, O.W. (2012). Deformation-based brain morphometry in rats. Neuroimage *63*, 47-53.

Huang, N., and Bonn, D. (2007). Viscosity of a dense suspension in Couette flow. J. Fluid Mech. *590*, 497-507.

Sugiyama, S., Saito, R., Funamoto, K., Nakayama, T., Sonoda, Y., Yamashita, Y., Inoue, T., Kumabe, T., Hayase, T., and Tominaga, T. (2013). Computational simulation of convection-enhanced drug delivery in the non-human primate brainstem: a simple model predicting the drug distribution. Neurol. Res. *35*, 773-781.

Tarbell, J.M., and Shi, Z.-D. (2013). Effect of the glycocalyx layer on transmission of interstitial flow shear stress to embedded cells. Biomech. Model Mechanobiol. *12*, 111–121.

Yao, W., Shen, Z., and Ding, G. (2013). Simulation of interstitial fluid flow in ligaments: comparison among Stokes, Brinkman and Darcy models. Int. J. Biol. Sci. *9*, 1050-1056.
